# Bovine neutrophil chemotaxis to *Listeria monocytogenes* in neurolisteriosis depends on microglia-released rather than bacterial factors

**DOI:** 10.1186/s12974-022-02653-1

**Published:** 2022-12-16

**Authors:** Stefano Bagatella, Neda Haghayegh Jahromi, Camille Monney, Margherita Polidori, Flavio Max Gall, Emma Marchionatti, Fabienne Serra, Rainer Riedl, Britta Engelhardt, Anna Oevermann

**Affiliations:** 1 Division of Neurological Sciences, Vetsuisse Faculty, University of Bern, Bremgartenstrasse 109a, CH-3012 Bern, Switzerland; 2 Graduate School for Cellular and Biomedical Sciences, University of Bern, Bern, Switzerland; 3 Theodor Kocher Institute (TKI), University of Bern, Bern, Switzerland; 4 Institute of Chemistry and Biotechnology, Competence Center for Drug Discovery, Zurich University of Applied Sciences (ZHAW), Wädenswil, Switzerland; 5 Clinic for Ruminants, Department of Clinical Veterinary Medicine, Vetsuisse Faculty, University of Bern, Bern, Switzerland; 6 Idexx Laboratory France, Saint Denis, France

**Keywords:** Neuroinfection, Neutrophils, Neurolisteriosis, Chemotaxis, Cattle, Microglia, IL-8, Formyl peptides

## Abstract

**Background:**

*Listeria monocytogenes* (*Lm*) is a bacterial pathogen of major concern for humans and ruminants due to its neuroinvasive potential and its ability to cause deadly encephalitis (neurolisteriosis). On one hand, polymorphonuclear neutrophils (PMN) are key players in the defense against *Lm*, but on the other hand intracerebral infiltration with PMN is associated with significant neural tissue damage. *Lm*-PMN interactions in neurolisteriosis are poorly investigated, and factors inducing PMN chemotaxis to infectious foci containing *Lm* in the central nervous system (CNS) remain unidentified.

**Methods:**

In this study, we assessed bovine PMN chemotaxis towards *Lm* and supernatants of infected endogenous brain cell populations in ex vivo chemotaxis assays, to identify chemotactic stimuli for PMN chemotaxis towards *Lm* in the brain. In addition, microglial secretion of IL-8 was assessed both ex vivo and in situ.

**Results:**

Our data show that neither *Lm* cell wall components nor intact bacteria elicit chemotaxis of bovine PMN ex vivo. Moreover, astrocytes and neural cells fail to induce bovine PMN chemotaxis upon infection. In contrast, supernatant from *Lm* infected microglia readily induced chemotaxis of bovine PMN. Microglial expression and secretion of IL-8 was identified during early *Lm* infection in vitro and in situ, although IL-8 blocking with a specific antibody could not abrogate PMN chemotaxis towards *Lm* infected microglial supernatant.

**Conclusions:**

These data provide evidence that host-derived rather than bacterial factors trigger PMN chemotaxis to bacterial foci in the CNS, that microglia have a primary role as initiators of bovine PMN chemotaxis into the brain during neurolisteriosis and that blockade of these factors could be a therapeutic target to limit intrathecal PMN chemotaxis and PMN associated damage in neurolisteriosis.

**Supplementary Information:**

The online version contains supplementary material available at 10.1186/s12974-022-02653-1.

## Introduction

Neurolisteriosis results from infection of the central nervous system (CNS) with *Listeria monocytogenes* (*Lm*), a Gram-positive bacillus infamous as potentially deadly food- and feed-borne pathogen in humans and animals. Upon ingestion, *Lm* is capable of colonizing the gastrointestinal tract and spreading to various target organs, causing gastroenteritis and invasive disease including septicemia, abortion and CNS infection [1, 2]. Notably, neurolisteriosis contributes to the high fatality rates associated with *Lm* infection, hence its ranking among the most lethal food-borne infections [3, 4]. In ruminants, neurolisteriosis is the most frequently encountered manifestation presenting as a selective brainstem encephalitis (rhombencephalitis), that may also occur in humans [5, 6]. Acute CNS lesions are characterized by the significant neuroparenchymal accumulation of polymorphonuclear neutrophils (PMN) in discrete foci known as microabscesses, cardinal pathological features of listeric rhombencephalitis [7, 8]. The topography of microabscesses with their alignment along axonal tracts and the frequent location of PMN in axonal spaces and myelin sheaths suggest that PMN migrate directionally towards bacteria located within axons [8, 9]. Severe PMN infiltration during early neurolisteriosis is associated with neuronal and axonal damage [7, 8, 10], providing evidence for bystander brain damage in the pathogenesis of neurolisteriosis. However, very few is known on dynamics, mechanisms and sequela of PMN chemotaxis to the CNS during infection [11, 12].

The integrity of functionally selective CNS barriers, amongst which the blood–brain barrier (BBB) is of utmost importance [13, 14], is crucial in regulating the intracerebral influx of immune cells including PMN [15, 16] and in preserving brain functions, as shown by the extensive cell death and cerebral edema occurring in neuroinflammatory conditions, which perturb BBB permeability [17–20]. PMN usually do not migrate through the healthy brain, but rather enter the CNS following BBB compromise and are generally regarded as marginal players in most intrathecal inflammatory diseases [21–24]. Significant PMN influx, however, is observed in certain pathological CNS conditions, such as cerebral ischemia [25, 26] and bacterial brain infections. In the latter, PMN exert a crucial role in bacterial phagocytosis and killing, but, on the other hand, can contribute to exacerbated immune responses via release of oxidants and proteolytic enzymes into the brain milieu causing collateral brain damage [27, 28]. The contribution of host and pathogen factors in PMN chemotaxis during bacterial brain infection remains largely unexplored. Resident brain cells are considered to play a role in intracerebral immune response against invading bacteria, as they can express pattern-recognition receptors (PRRs), such as Toll-like receptors, that are able to recognize pathogen-associated molecular patterns (PAMPs), and secrete cytokines and chemokines that can enhance PMN transmigration across the BBB [29–34]. Bacterial virulence factors might also contribute to intracerebral PMN recruitment, as shown for β-hemolysin in streptococcal meningitis [35], but roles for additional bacterial components in PMN chemotaxis into the infected brain remain yet poorly defined. Few studies indicated that *Lm* formyl peptides could induce murine PMN chemotaxis in vitro and suggested that they may be involved in PMN recruitment into the liver of infected mice [36, 37], but their role in PMN chemotaxis during CNS infection, together with other *Lm* factors, is completely unknown.

In this study, we sought to identify bacterial and host factors responsible for eliciting PMN chemotaxis to *Lm* in the brain. To achieve so, we assessed the chemotactic effect of *Lm* and of infected endogenous brain cells towards bovine PMN through ex vivo chemotaxis assays. We chose a bovine model system as cattle are relevant natural hosts of *Lm* in contrast to mice. Our data show that PMN chemotaxis is independent from *Lm* or *Lm*-derived factors. Moreover, amongst endogenous brain cells supernatants from infected bovine astrocytes and neuronal cells failed to elicit PMN chemotaxis. Infected microglia, on the other hand, induced significant chemotaxis of bovine PMN, suggesting that microglia play a central role in orchestrating PMN chemotaxis during neurolisteriosis. Moreover, microglia expressed and secreted IL-8 in vitro and in situ within very early lesions. However, treatment of microglial supernatant with anti-IL-8 antibody did not prevent PMN chemotaxis, suggesting that microglia release additional chemotactic factors that contribute to PMN chemotaxis in neurolisteriosis.

## Materials and methods

### Bovine PMN isolation

Blood was collected from cows that were venipunctured for diagnostic purposes at the Ruminant Clinic of the Vetsuisse Faculty, University of Bern (Switzerland). Donor cows were hospitalized for treatment of non-infectious conditions or localized inflammations/infections. Available clinical data on donor cows are given in Additional file 8: Table S1. Ethical approval for this study (number: BE33/17) was obtained from the Ethics Commission of the Canton of Bern.

PMN were isolated as described previously [38] with slight modifications. Briefly, blood was collected aseptically by jugular venipuncture into an equal volume of Alsever’s solution, distributed into conical 50 ml tubes and centrifuged at 1000×*g* for 20 min at 4 °C. The plasma, buffy coat and the upper third of the red cell pack were discarded and the red cell pack was resuspended in up to 35 ml ice-cold phosphate-buffered saline (PBS). The red cell pack suspension was gently overlaid on top of 10 ml of Ficoll–Paque PREMIUM 1.084 (GE Healthcare, Chicago, IL) and centrifuged at 400×*g* for 40 min at RT, after which the supernatant containing residual mononuclear cells was discarded. The red cell pack was resuspended in hypotonic lysis buffer (8.29 g/l NH_4_Cl, 1 gl NaHCO_3_, pH 7.4) to lyse residual erythrocytes and centrifuged at 800×*g* for 5 min at 4 °C. Then, the supernatant was discarded, and the pellet resuspended a second time in 10 ml of lysis buffer followed by centrifugation at 600×*g* for 5 min at 4 °C. The purified PMN pellet was washed twice in ice-cold PBS and centrifuged at 400×*g* for 5 min at 4 °C, after which PMN were resuspended in PBS for further use.

### Staining and preparation of bovine PMN

Before use, PMN were stained with CellTracker Green CMFDA (CTG, 0.5 μM; Invitrogen) and LIVE/DEAD fixable Near-IR Dead Cell Stain (LD, 1:300; Invitrogen) in PBS for 30 min at 37 °C, 5% CO_2_. PMN were washed twice and resuspended in chemotaxis assay medium (CAM) containing Dulbecco's modified Eagle's medium (DMEM; low glucose, pyruvate, no glutamine, no phenol red; Thermo Fisher Scientific) adjusted to pH 6.8 and supplemented with 10% fetal calf serum (FCS; Bioswisstec, Schaffhausen, Switzerland) and 1% l-Glutamine 0.2 M (Merck Millipore, Darmstadt, Germany). PMN number and viability were assessed using a CASY Cell Counter (OLS OMNI Life Science, Bremen, Germany) before PMN concentration was adjusted for each assay.

### Culture of primary cells and cell lines, and immunophenotypic characterization of primary cells

Primary bovine astrocytes were isolated from fresh cattle brains collected at local slaughterhouses adopting a previously described method [39] with slight modifications. Briefly, cerebral cortex was mechanically dissociated and enzymatically digested with collagenase Type I (final concentration 500 µg/ml; Sigma, C0130), after which cells were isolated through repeated bovine serum albumin (BSA, 25%; Sigma #A2153) density gradient separations and sequentially passed through 100, 70 and 30 µM nylon screens. Cells were seeded in flasks and grown to ~ 80% confluency, after which they were trypsinized, passaged 1:1 and allowed to adhere for 30 min. This selective attachment, in which slow-adhering cells were collected and discarded, allowed for enrichment of fast-adhering astrocytes, as previously reported [39]. Astrocytes were seeded in poly-l-lysine-coated 24-well plates (Corning, Vitaris, Baar, Switzerland) in complete medium (DMEM-FCS) composed of DMEM supplemented with 10% FCS, 1% l-Glutamine 0.2 M and 0.5% gentamicin and grown to > 90% confluence. For immunophenotypic characterization, primary bovine astrocytes grown on poly-l-lysine-coated 12 mm glass coverslips were fixed with 4% paraformaldehyde (PFA, Sigma-Aldrich) for 10 min at RT. Methods, primary and secondary antibodies used are indicated in Additional file 9: Table S2. Cell imaging was carried out using an Olympus Fluoview FV3000 confocal laser scanning microscope (Olympus, Tokyo, Japan), equipped with 405‐nm, 488‐nm and 561-nm laser channels. Obtained images were processed using the open-source software Fiji [40]. The percentage of GFAP, vimentin, S100 and Iba-1 immunolabeled cells, respectively, was evaluated in two–three random fields of view (FOV) from 6 independent experiments (17 FOV in total) at 20× magnification.

Fetal bovine brain cells (FBBC-1) [41] were grown in flasks in a 1:1 mixture of DMEM and Ham’s F-12 medium (DMEM/F12, Life Technologies) supplemented with 10% FCS, 50 ng/ml epithelial growth factor, 50 ng/ml bFGF, 100 U/ml penicillin, 10 μg/ml streptomycin, and 0.5% N2 supplement (Life Technologies). After reaching confluence, FBBC-1 cells were trypsinized and reseeded into 24-well plates with DMEM supplemented with 10% FCS without penicillin/streptomycin, grown to confluence and then incubated with 100 μM forskolin (Merck‐Millipore, Schaffhausen, Switzerland) to induce neuronal differentiation 18 h prior to infection.

Primary bovine microglial cells were isolated by mechanical dissociation and Percoll (Cat. No. P1644, Sigma) gradient centrifugation, and were cultured as recently described [42]. As assessed through recently described characterization methods [42], almost pure bovine microglial cultures were obtained.

### Bacterial strains and cultures

*Lm* strain JF5203 (lineage I, clonal complex 1, sequence type 1, https://www.ncbi.nlm.nih.gov/nuccore/NZ_LT985474.1) isolated from a bovine rhombencephalitis case was used as the wild-type (WT-*Lm*) reference strain in our experiments. An isogenic deletion mutant for the *hly* gene (Δ*hly*) generated for this study (Additional file 6: Methods S1) and the EGD-e strain (lineage II, clonal complex 9) were used to compare the effect of bacterial listeriolysin-O (LLO) deficiency and lineage-related differences on PMN chemotaxis, respectively.

Single bacterial colonies were inoculated in brain–heart infusion (BHI) broth and grown overnight at 37 °C on a shaking platform. The day of the experiment, bacteria were pelleted by centrifugation (2100×*g*, for 5 min) and washed once in PBS, after which they were diluted in PBS to an OD_600_ of 0.6 (equivalent to ~ 10^9^ CFU/ml) then further diluted in CAM to a final concentration of 6 × 10^5^ CFU/well. Bacterial concentration was retrospectively assessed for each experiment by CFU enumeration following overnight incubation on BHI-agar plates.

Where indicated, pelleted bacteria were opsonized in 10% pooled bovine serum in PBS for 30 min at 37 °C, then washed twice in PBS prior to being diluted in CAM. In selected experiments, where alive bacteria were used, the medium from the upper compartment was collected and plated on BHI-agar for CFU counting, to assess bacterial migration into the upper chamber. As a high number of resuspended *Lm* were observed to migrate into the upper compartment, thus possibly diluting the chemotactic gradient, we also resuspended bacteria in an agarose solution (final concentration 6.5 mg/ml, Sigma-Aldrich) to a dilution of 6 × 10^7^ CFU/ml, of which 10 μl was plated on the bottom of a 24-well plate and, once solidified, further sealed with 10 μl of agarose solution. CAM was added on top of the agarose drops at the final volume required for the assay, bringing the final concentration of bacteria per well to 6 × 10^5^ CFU/well. To produce heat-killed *Lm* (HK-*Lm*), bacteria were incubated at 60 °C for 30 min and loss of viability was checked by lack of overnight growth after plating. Bacterial culture supernatants were obtained by inoculating *Lm* in CAM, after which bacteria were grown overnight at 37 °C on a rocking platform. The following day, the supernatant was separated from bacteria after centrifugation, filter-sterilized through a 0.22 μm pore size syringe filter (Millipore, Switzerland) and assessed for sterility by absence of CFU after plating. Culture supernatants were stored at − 20 °C and finally used for the experiments after being diluted 1:10 in fresh CAM.

### Infection of endogenous brain cells and preparation of culture supernatants

To obtain culture supernatant from infected astrocytes, FBBC-1 and microglia, the day prior to the assay wells were washed 3× with sterile PBS and fresh CAM without antibiotics was added. The following day, cells were inoculated with WT-*Lm* at the equivalent of a multiplicity of infection (MOI) of 10 in FCS-free CAM, and plates were centrifuged at 300×*g* for 5 min to synchronize infection. Cells were incubated with bacteria for 2 h, the medium was discarded, and cells were washed 3× in PBS to remove extracellular bacteria. Fresh CAM with 0.01 mg/ml gentamicin was added to the wells to kill residual extracellular adhering bacteria and prevent *Lm* rapid replication inside the medium. Cell culture supernatant was collected following 4 h of infection, filter-sterilized and stored at -20 °C until the day of the experiments. As negative control, supernatants of mock-infected cells undergoing the same experimental procedures were used. In selected experiments, supernatants from infected microglia were pre-incubated for 1 h at 4 °C with a mouse monoclonal anti-sheep IL-8 antibody (final concentration 50 μg/ml, MCA1660, clone 8M6, Bio-Rad) before testing in the chemotaxis assay. To confirm a specific blocking effect of the anti-IL-8 antibody, the same procedures were also repeated on medium containing bovine recombinant IL-8 (Bovine IL-8, 50 ng/ml; Kingfisher Biotech, Inc. St. Paul, MN, USA), while bovine IL-8-containing medium incubated with Mouse IgG1 (MoIgG1, final concentration 50 µg/ml; MAB002, R&D systems) was used as negative control.

### Transwell chemotaxis assay

PMN chemotaxis was assessed in 24-well Transwell plates with 5.0 µm size pore polycarbonate membrane inserts (# 3421, Corning). Membranes were coated with rat-tail collagen prepared according to the method of Bornstein [43], following a previously described procedure [44]. The lower compartment of the Transwell chamber was filled with 600 μl of unstimulated CAM to assess random migration, or with CAM containing bacteria, chemotactic factors, or cell supernatants (Additional file 10: Table S3). Following preliminary tests and titrations, bovine IL-8 at 50 ng/ml was used as a positive control for bovine PMN chemotaxis. Following isolation, PMN were incubated for 30 min at 37 °C, 5% CO_2_ to allow them to recover. Then, 5 × 10^5^ cells suspended in 100 µl CAM were added to the upper compartment and the assay was started by assembling the Transwell plate. Migration was allowed to occur for 1 h at 37 °C, 5% CO_2_, and migrated PMN were detached from the lower-side of the filter by incubating the plate 15 min at 4 °C following addition of 60 μl 0.5 M EDTA to the bottom chamber, as previously described [45]. The plate was then gently shaken, the PMN-containing medium was collected from the lower compartment and migrated cells were quantified with an Attune NxT Flow Cytometer (Thermo Fisher Scientific, Switzerland). Data were analyzed using FlowJoTM software (Tree Star, Ashland, OR, USA). PMN purity was on average 90% according to FSC and SSC gating. PMN gating strategy was based on control stainings as illustrated in Additional file 1: Fig. S1. Briefly, PMN were gated according to the FSC and SSC localization of cells positive for the anti-bovine granulocyte monoclonal antibody CH138A (IgG1, Cat. No WS0608B-100, Kingfisher Biotech, Inc.), as illustrated in Additional file 2: Fig. S2. PMN staining procedures with CH138A are indicated in Additional file 7: Methods S2. After excluding debris and contaminants with the FSC vs SSC gating, doublets and aggregates were excluded on an FSC-A vs FSC-H plot. The number of migrated PMN per well was enumerated by gating on viable and CTG-positive cells.

As the number of intact PMN decreased during incubation from the initial 5 × 10^5^ PMN in 100 µl (average: 3.7 × 10^5^ PMN in 100 µl), we inoculated 100 μl of 5 × 10^5^ PMN in 500 μl of CAM and incubated it for 1 h as control for the reduction of PMN number over the course of the incubation. The number of cells recovered from this sample corresponds to the pool of PMN inoculated into the upper well that would have been able to migrate into the lower well (termed “input”). To allow comparison between different experiments, the absolute number of PMN per well was normalized to an input of 5 × 10^5^ PMN in each experiment and the number of migrated PMN was expressed as absolute cell number normalized to the input.

In preliminary experiments assessing the dose effect of bovine IL-8 on PMN chemotaxis (Additional file 4: Fig. S4), PMN collected from the lower well were stained with Trypan blue and viable PMN were enumerated with a hemocytometer. The number of viable PMN migrated in the lower well in experiments assessing the blocking effect of the anti-IL-8 antibody against bovine recombinant IL-8 (Fig. 4C) was assessed with a CASY counter.

### ELISA for quantification of bovine IL-8 in microglia supernatant

Secretion of bovine IL-8 in the supernatant of both infected and non-infected microglia was assessed through a sandwich enzyme-linked immunosorbent assays (ELISA) similarly as previously reported [46] with slight modifications. Briefly, 96-well plates (Corning, Vitaris, Baar, Switzerland) were coated with 5 µg/ml of mouse anti-sheep IL-8 antibody (MCA1660, clone 8M6, Bio-Rad) and incubated overnight at 4 °C. Wells were then washed and incubated with supernatants from infected microglia, and non-infected microglia or bovine IL-8 (Kingfisher Biotech, Inc. St. Paul, MN, USA), which was applied at different concentrations to prepare the standard curve, for 3 h at RT. After washing, wells were incubated with a rabbit anti-sheep IL-8 antibody (AHP425, 1:500; Bio-Rad) for 4 h at RT, then plates were washed, and detection was carried out using the Thermo Scientific TMB QUICK Liquid Substrate for ELISA (10748352, Fisher Scientific). Reactions were stopped by addition of H_2_SO_4_ and plates were read at 450 nm using a Cytation 5 imaging multimode reader (BioTek). IL-8 concentration in microglial supernatant samples was calculated in the Gen5 v3.0 software (BioTek) based on the OD values and concentrations of the standard curve.

### In situ immunohistochemistry and immunofluorescence of bovine listeriosis cases

To assess microglial IL-8 expression in acute lesions of naturally infected cattle, formalin-fixed paraffin-embedded (FFPE) sections of brains from listeriosis cases were processed for immunohistochemistry (IHC) and immunofluorescence (IF).

Following screening of archive neurolisteriosis cases for the presence of acute microabscesses, classified as such according to previously published histologic criteria [8], 9 neurolisteriosis cases were selected for immunohistochemical processing. Of such cases, 5 were further selected for IF analysis following the assessment of marked IL-8 positivity centered on microabscesses on IHC.

For both IHC and IF, dewaxing, antigen retrieval (citrate buffer, pH 6) and blocking with 5% normal goat serum (NGS) were performed as previously described [42].

For IHC, sections were incubated overnight at 4 °C in PBS-Tween (PBS-T) containing 10% NGS and mouse anti-sheep IL-8 antibody (1:1000, MCA1660, clone 8M6, Bio-Rad). Signal detection was carried out using the Mouse and Rabbit Specific HRP/DAB (ABC) Detection IHC kit (ab64264; Abcam plc, Cambridge, UK) according to manufacturer’s instructions. Slides were then counterstained with Mayer's hemalum solution (Merck KGaA, Darmstadt, Germany), mounted with Aquatex (Merck KGaA) and finally imaged with a Zeiss microscope. As negative control, neurolisteriosis tissue was subjected to the same protocol using unspecific mouse IgG instead of the specific IL-8 antibody. As positive control, neurolisteriosis tissue containing numerous PMN was used.

For IF, incubation with rabbit anti-P2RY12 (1:200, 55043A, AnaSpec), rabbit anti-Iba1 (1:500, 013-27593, Wako) and/or mouse Factor XIII A (F13A1, 1:100, MA5-11751, Thermo Fisher) were performed as previously described [42]. In addition, sections were incubated with mouse anti-sheep IL-8 antibody (1:400, MCA1660, clone 8M6, Bio-Rad) overnight at 4 °C, following antigen retrieval in Target Retrieval solution, pH 9 (S2367, Agilent Technologies) for 15 min at 95 °C in a H2850 Microwave Processor (EBSciences, East Granby, Hartford County, Connecticut). Labeling was visualized by 1 h incubation at RT with secondary antibodies coupled to Alexa Fluor 555 or Alexa-Fluor 647 (Invitrogen), diluted at either 1:400 (P2RY12, Iba1 and F13A1) or 1:100 (IL-8). Moreover, staining for *Lm* was performed using a rabbit anti-Listeria polyclonal antibody (1:200, Difco Laboratories, Detroit MI, USA) pre-labeled with the Zenon Alexa Fluor 488 Rabbit IgG Labeling Kit (Z23502, Invitrogen) according to the manufacturer’s instructions. As negative control, neurolisteriosis tissue was subjected to the same protocol using unspecific mouse IgG instead of the specific IL-8 antibody. As positive control, neurolisteriosis tissue containing numerous PMN was used.

All tissue slides were additionally stained with DAPI (1:1000, Invitrogen). Finally, slides were mounted with coverslips using glycergel (Agilent) and Z-stack images were captured using an Olympus Fluoview FV3000 confocal laser scanning microscope (Olympus, Tokyo, Japan). Images were processed with the software Fiji [40].

### Statistical analysis

PMN chemotaxis was evaluated in a minimum of three independent experiments performed in triplicates and conducted with PMN from a minimum of three different individual donor cows for each condition tested (with the exception of IL-8 blocking and IL-8 dose effect assays which were conducted in duplicates or triplicates with PMN from one to three donor cows). PMN chemotaxis rates displayed variability depending on the donor animal. Therefore, data from chemotaxis experiments are represented as superimposed scatter dot plots highlighting donor cows, from which PMN were extracted. Statistical analysis was carried out using GraphPad Prism v.9 (GraphPad Software, CA, USA). Nonparametric Mann–Whitney *U* tests were performed in each assay by comparing PMN chemotaxis in tested conditions with random migration towards medium alone. Mann–Whitney *U* tests were also conducted to compare CFU collected from the upper well. Differences were considered statistically significant at *P* < 0.05 and *P* values are indicated in the figures by asterisks: *P < 0.05; **P < 0.01; ***P < 0.001; ****P < 0.0001. Non-significant differences are indicated in the figures as “ns”.

## Results

### Formyl peptides, *Lm* and *Lm* factors do not elicit bovine PMN chemotaxis

**Fig. 1:**
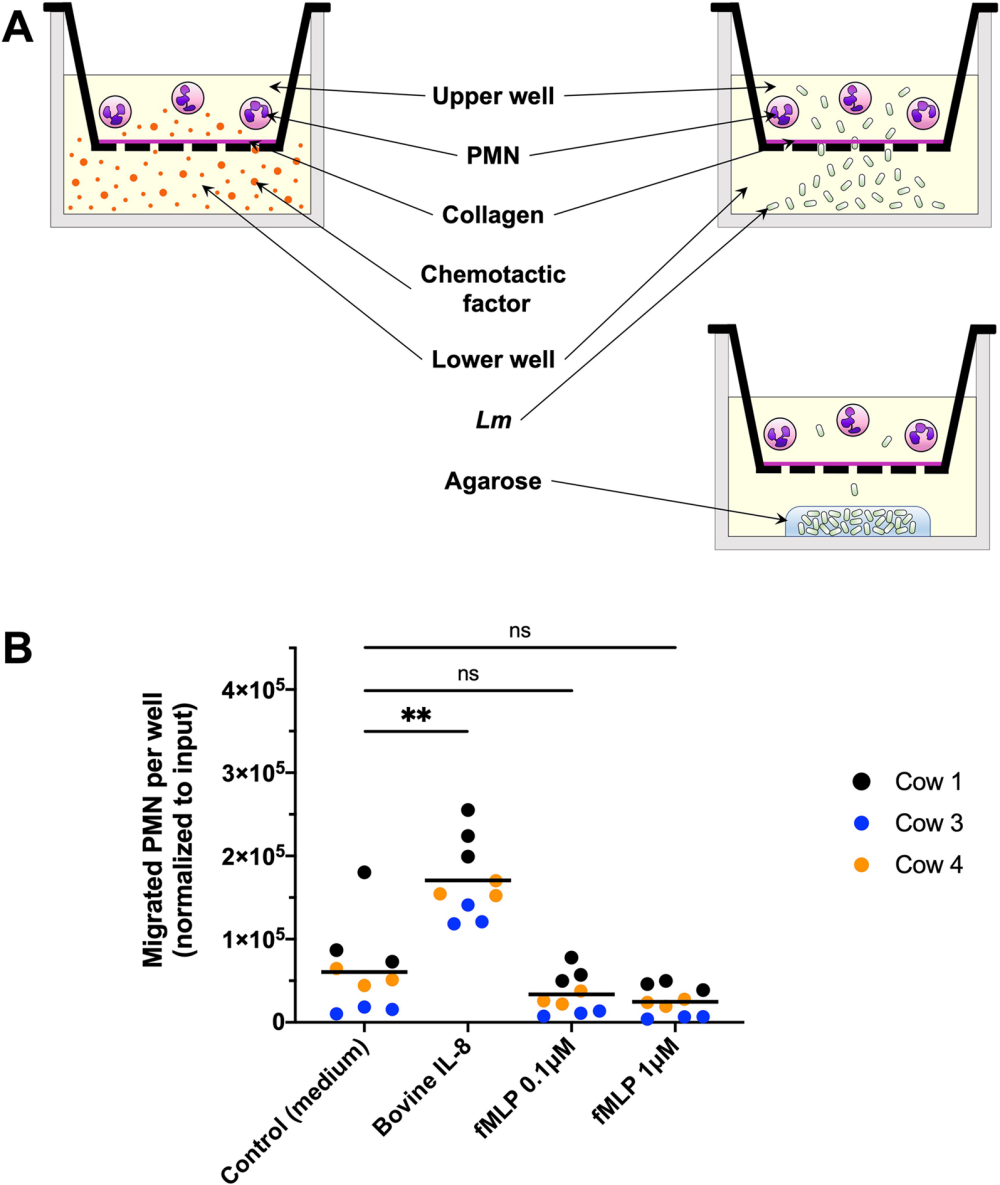
Bovine PMN are responsive to IL-8, but unresponsive to fMLP in the chemotaxis assay. **A** Schematic representation of the chemotaxis assay. Bovine PMN are loaded into the upper chamber of a Transwell inset and are allowed to migrate through the porous bottom membrane toward the lower chamber containing chemotactic stimuli (left) or whole *Lm*, either free in medium (upper right) or trapped under a layer of agarose (lower right). Free bacteria move into the upper chamber, while the agarose layer limits *Lm* access to the upper chamber (see also Fig. 2C).** B** PMN chemotaxis toward medium alone (control, random migration), bovine IL-8 (positive control), and fMLP at a concentration of 0.1 and 1 µM. Data are represented as means on a superimposed scatter dot plot of 3 independent experiments, each performed in triplicates. Data are color-coded according to the donor cows, from which PMN were extracted. Statistical analyses: Mann–Whitney *U* tests (**P < 0.01; *ns* non-significant)

Previous work from our group showed that PMN localize with bacteria and appear to track intracerebral *Lm* during neurolisteriosis in ruminants [8, 9]. Therefore, we hypothesized that bovine PMN are attracted to infectious foci by *Lm*-derived factors including formyl peptides. To assess the contribution of bacterial factors to bovine PMN chemotaxis, we set up an ex vivo chemotaxis assay, in which we assessed the chemotaxis of freshly isolated bovine PMN from an upper chamber across a filter membrane into a lower compartment in a Transwell system containing either *Lm*-derived factors or whole bacteria (Fig. 1A). Recovery of migrated bovine PMN was only possible following addition of EDTA into the medium of the lower well, as we noticed that bovine PMN, which had migrated across the Transwell membrane, firmly adhered to the lower side of the filter insert and had to be actively detached for collection and counting (own observations). To select an effective positive control for PMN chemotaxis, we tested bovine PMN chemotaxis towards bovine IL-8 and fMLP, using, for the latter, concentrations reported to efficiently elicit chemotaxis of human and murine PMN (i.e., 0.1 and 1 µM) [45, 47, 48]. While bovine IL-8 has been shown to strongly elicit bovine PMN chemotaxis [49], studies on the chemotactic effect of fMLP are contradictive with some indicating that bovine PMN do not migrate towards formyl peptides [50–52], while others claim a chemotactic effect of fMLP on bovine PMN [53–55]. As illustrated in Fig. 1B, neither of the tested fMLP concentrations could induce significant chemotaxis of bovine PMN. On the other hand, bovine IL-8 elicited significant PMN chemotaxis (Fig. 1B) and was, therefore, selected as positive control for bovine PMN chemotaxis.

**Fig. 2:**
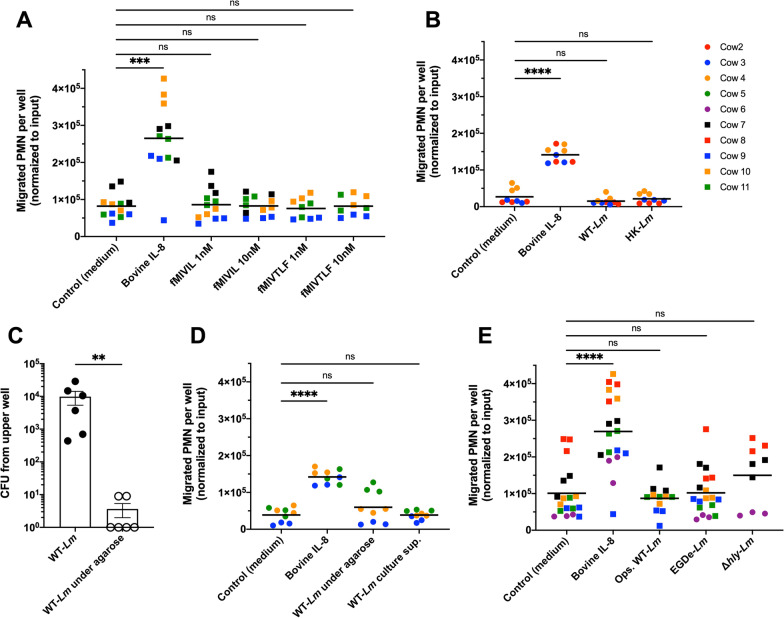
Bovine PMN are unresponsive to *Lm* formyl peptides, *Lm* factors released in medium and intact *Lm*. Data points are color-coded according to the PMN donor cows, as listed in the panel on the upper right. Statistical analyses: Mann–Whitney *U* tests (**P < 0.01; ***P < 0.001; ****P < 0.0001; *ns* non-significant). **A** PMN chemotaxis toward *Lm* formyl peptides (fMIVIL and fMIVTLF), both tested at concentrations of 1 and 10 nM, in comparison with medium alone and bovine IL-8. Data are represented as means on a superimposed scatter dot plot of 3 independent experiments, each performed in triplicates. **B** PMN chemotaxis toward WT-*Lm* suspended in medium (WT-*Lm*) and heat-killed WT-*Lm* suspended in medium (HK-*Lm*), in comparison with PMN chemotaxis toward medium alone and bovine IL-8. Data are represented as means on a superimposed scatter dot plot of 3 independent experiments, each performed in triplicates. **C** Comparison of CFU collected from the upper well of WT-*Lm* freely suspended in medium (WT-*Lm*, black dots) with WT-*Lm* trapped under agarose (WT-*Lm* under agarose, white dots). Values are normalized to an inoculum of 10^5^ CFU to allow comparison between experiments. Data are represented as mean ± SEM (standard error of mean) of 2 independent experiments performed in triplicates.** D** PMN chemotaxis toward WT-*Lm* trapped under agarose and WT-*Lm* culture supernatant diluted 1:10 in fresh CAM (WT-*Lm* culture sup.) in comparison with medium alone and bovine IL-8. Data are represented as means on a superimposed scatter dot plot of 3 independent experiments, each performed in triplicates. **E** PMN chemotaxis toward WT-*Lm* opsonized with bovine serum and freely resuspended in medium (Ops. WT-*Lm*, *n* = 4), EGDe-*Lm* freely resuspended in medium (EGDe-*Lm*, *n* = 6) and Δ*hly-Lm* freely resuspended in medium (Δ*hly-Lm*, *n* = 3) in comparison with medium alone (*n* = 6) and bovine IL-8 (*n* = 6). Data are represented as means on a superimposed scatter dot plot of 3 to 6 independent experiments, each performed in triplicates. For conditions tested in less than 6 animals, statistical analysis was performed between each condition tested and corresponding donor cows used to assess chemotaxis toward medium alone

We first assessed bovine PMN chemotaxis towards *Lm*-derived formyl peptides, which were reported to induce chemotaxis of murine PMN more efficiently and at lower concentrations than fMLP [37]. As illustrated in Fig. 2A, neither fMIVIL nor fMIVTLF, which were previously reported to be potent chemotactic agents and FPR agonists for murine PMN and human PMN-like cells [37, 56], could elicit chemotaxis of bovine PMN at any concentration tested, indicating that, unlike murine PMN, bovine PMN do not chemotactically respond to those formyl peptides.

As *Lm* formyl peptides failed to induce chemotaxis of bovine PMN, we next investigated the chemotactic effect of intact/whole bacteria. Chemotaxis of bovine PMN could not be induced by exposure to either viable or heat-killed WT-*Lm* (Fig. 2B), suggesting that whole bacteria do not elicit bovine PMN chemotaxis regardless of their viability. However, viable *Lm* were capable of actively migrating into the upper well towards PMN within 1 h of incubation (Figs. 1A, 2C) potentially diluting the chemotactic gradient necessary for inducing PMN chemotaxis into the lower well. Therefore, we sought to prevent bacteria from freely migrating into the upper well by trapping WT-*Lm* under a layer of agarose (Fig. 1A). Efficient inhibition of bacterial migration was confirmed by a > 2 Log reduction in CFU cultured from the upper well (Fig. 2C). Similar to freely suspended *Lm*, agarose-captured *Lm* failed to induce bovine PMN chemotaxis (Fig. 2D), indicating that the lack of PMN chemotaxis was unrelated to the abrogation of a chemotactic gradient.

Similarly, supernatants (diluted 1:10) of *Lm* cultured overnight failed to attract bovine PMN (Fig. 2D).

*Lm* opsonization with serum factors is considered to be crucial for efficient bacterial recognition and phagocytosis [57, 58], particularly when PMN are suspended in medium [59]. Therefore, as our chemotaxis assay was conducted with PMN in suspension, we tested whether opsonization of *Lm* with serum would enhance bovine PMN chemotaxis. As shown in Fig. 2E, opsonization of WT-*Lm* did not enhance bovine PMN chemotaxis.

Evidence of a chemotactic effect of *Lm *in vivo was previously provided in mice infected with a *Lm* reference strain belonging to lineage II (i.e., EGD) [36]. As *Lm* genetic heterogeneity has been associated with variation in virulence [60], we investigated bovine PMN chemotaxis towards the *Lm* laboratory reference strain EGDe. Like our WT-*Lm* (belonging to phylogenetic lineage I), the EGDe strain (belonging to lineage II) did not induce significant chemotaxis of bovine PMN (Fig. 2E), indicating that the lack of chemotactic effect towards *Lm* was not attributable to the phylogenetic lineage of our WT-*Lm* strain.

Finally, to exclude that the *Lm*-derived cholesterol-dependent cytolysin (CDC) listeriolysin O (LLO), encoded by *hly*, inhibits PMN chemotaxis as it has been shown for other bacterial CDCs [61, 62], we assessed chemotaxis towards the *Lm* Δ*hly* deletion mutant. As shown in Fig. 2E, lack of LLO expression did not significantly enhance bovine PMN chemotaxis towards *Lm*, suggesting that PMN chemotaxis is not abrogated by bacterial LLO secretion.

Overall, these data show that bovine PMN are unresponsive to formyl peptides, intact *Lm* and *Lm* products ex vivo, suggesting that bovine PMN chemotaxis is not directly elicited by bacterial factors but involves host factors.

### Bovine PMN chemotaxis is induced by supernatant from infected microglia, but not from infected astrocytes or neuronal cells

Due to bovine PMN unresponsiveness to *Lm*-derived stimuli, we tested in the same ex vivo system whether endogenous CNS cell populations release chemotactic factors for PMN during infection. To assess the contribution of infected neural cells on bovine PMN chemotaxis, we collected supernatants from primary bovine astrocytes, FBBC-1 cells and primary bovine microglia infected with *Lm* at an MOI of 10 and tested their chemotactic effect on bovine PMN.

The phenotype of primary bovine astrocytes was determined by IF (Additional file 3: Fig. S3). Similar to previously reported data [63], the vast majority of cultured cells stained for GFAP (95.53% ± 2.37 SD), confirming their astrocytic lineage, and Vimentin (98.56% ± 0.93 SD), with only few Iba1-positive cells (0.68% ± 0.51 SD) identifiable as contaminating microglia. For primary microglial cell cultures, and as previously characterized [42], approximately 100% of cells expressed Iba1 (not shown) indicating high microglial purity.

**Fig. 3:**
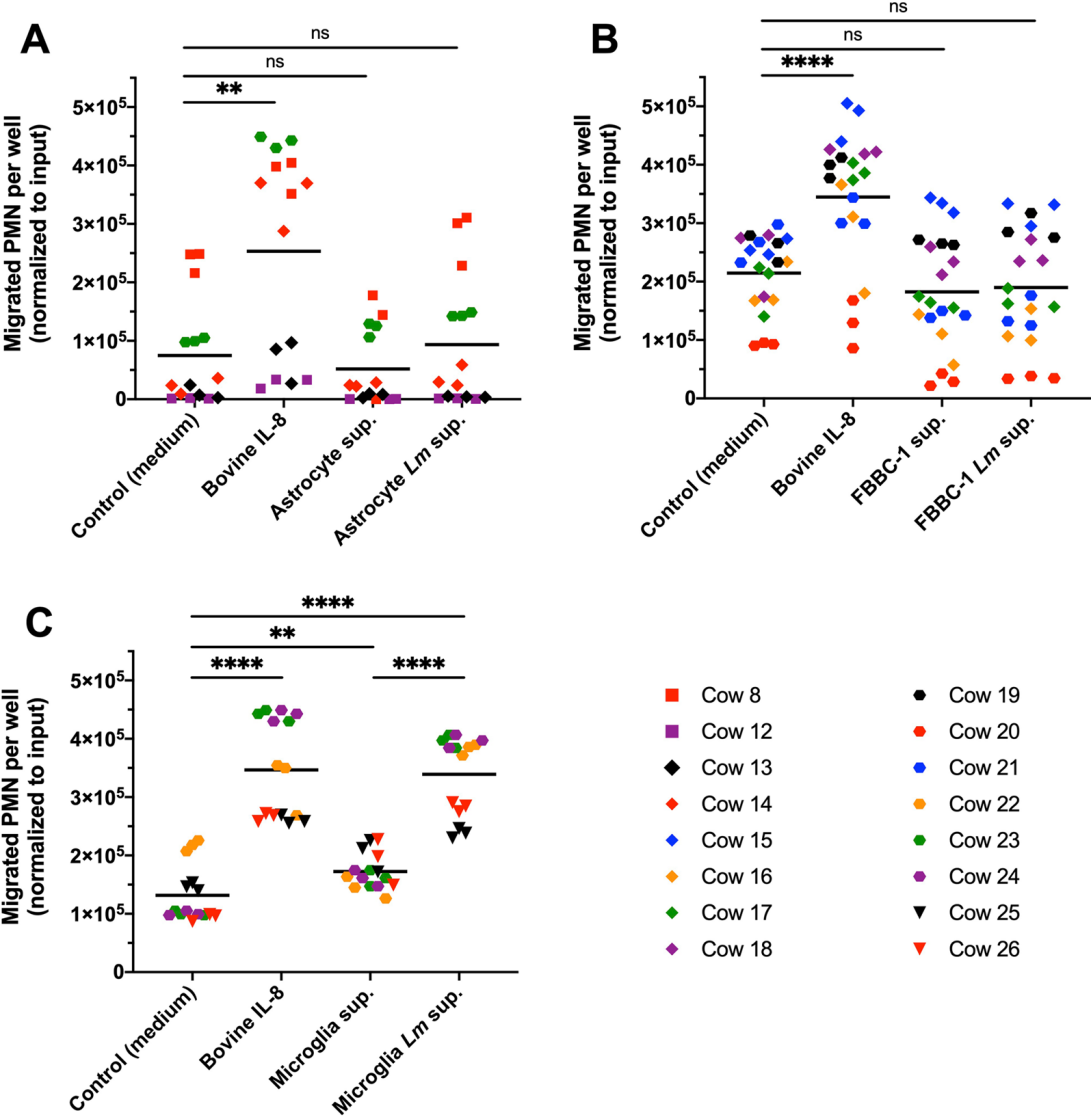
Supernatant from infected microglia, but not from infected astrocytes or FBBC-1 cells, induces bovine PMN chemotaxis. Donor cows from which PMN were extracted to conduct chemotaxis assays in each represented experiment are indicated in two columns in the lower right panel. Statistical analyses: Mann–Whitney *U* tests (**P < 0.01; ****P < 0.0001; *ns* non-significant). **A** PMN chemotaxis toward astrocyte-conditioned medium (Astrocyte sup.) and medium from astrocytes infected with WT-*Lm* (Astrocyte *Lm* sup.), in comparison with medium alone and bovine IL-8. Data are represented as means on a superimposed scatter dot plot of 5 independent experiments, each performed in triplicates. **B** PMN chemotaxis toward FBBC-1-conditioned medium (FBBC-1 sup.) and medium from FBBC-1 infected with WT-*Lm* (FBBC-1 *Lm* sup.), in comparison with medium alone and bovine IL-8. Data are represented as means on a superimposed scatter dot plot of 7 independent experiments, each performed in triplicates. **C** PMN chemotaxis toward microglia-conditioned medium (Microglia sup.) and medium from microglia infected with WT-*Lm* (Microglia *Lm* sup.), in comparison with medium alone and bovine IL-8. Microglia-conditioned medium exerts modest chemotaxis on bovine PMN, and the chemotactic effect increased upon infection of microglia. Data are represented as means on a superimposed scatter dot plot of 5 independent experiments, each performed in triplicates

Medium from non-infected astrocytes did not induce PMN chemotaxis, as shown in Fig. 3A. Surprisingly, supernatant from infected astrocytes also failed to elicit PMN chemotaxis (Fig. 3A). Similarly, neither uninfected nor *Lm*-infected supernatants from FBBC-1 cells previously differentiated into neurons were capable of eliciting PMN chemotaxis (Fig. 3B), suggesting that infected astrocytes and neuronal cells do not release chemoattractive molecules for bovine PMN. In contrast, supernatant from *Lm*-infected microglia was strongly chemotactic for bovine PMN as it induced PMN chemotaxis rates similar to those elicited by bovine IL-8 (Fig. 3C). Interestingly, supernatant from non-infected microglia could also elicit modest PMN chemotaxis, albeit at significantly lower levels than *Lm*-infected microglia supernatant (Fig. 3D), giving further support to transcriptomic data indicating activation of bovine microglia following isolation and cell culture [42]. Taken together, these data indicate that *Lm*-infected microglia release factors chemotactic for bovine PMN, suggesting that microglial cells are the major players in PMN recruitment to infectious foci during neurolisteriosis.

### Bovine microglia express IL-8 upon *Lm* infection both in vitro and in situ, but IL-8 blocking does not prevent bovine PMN chemotaxis

**Fig. 4:**
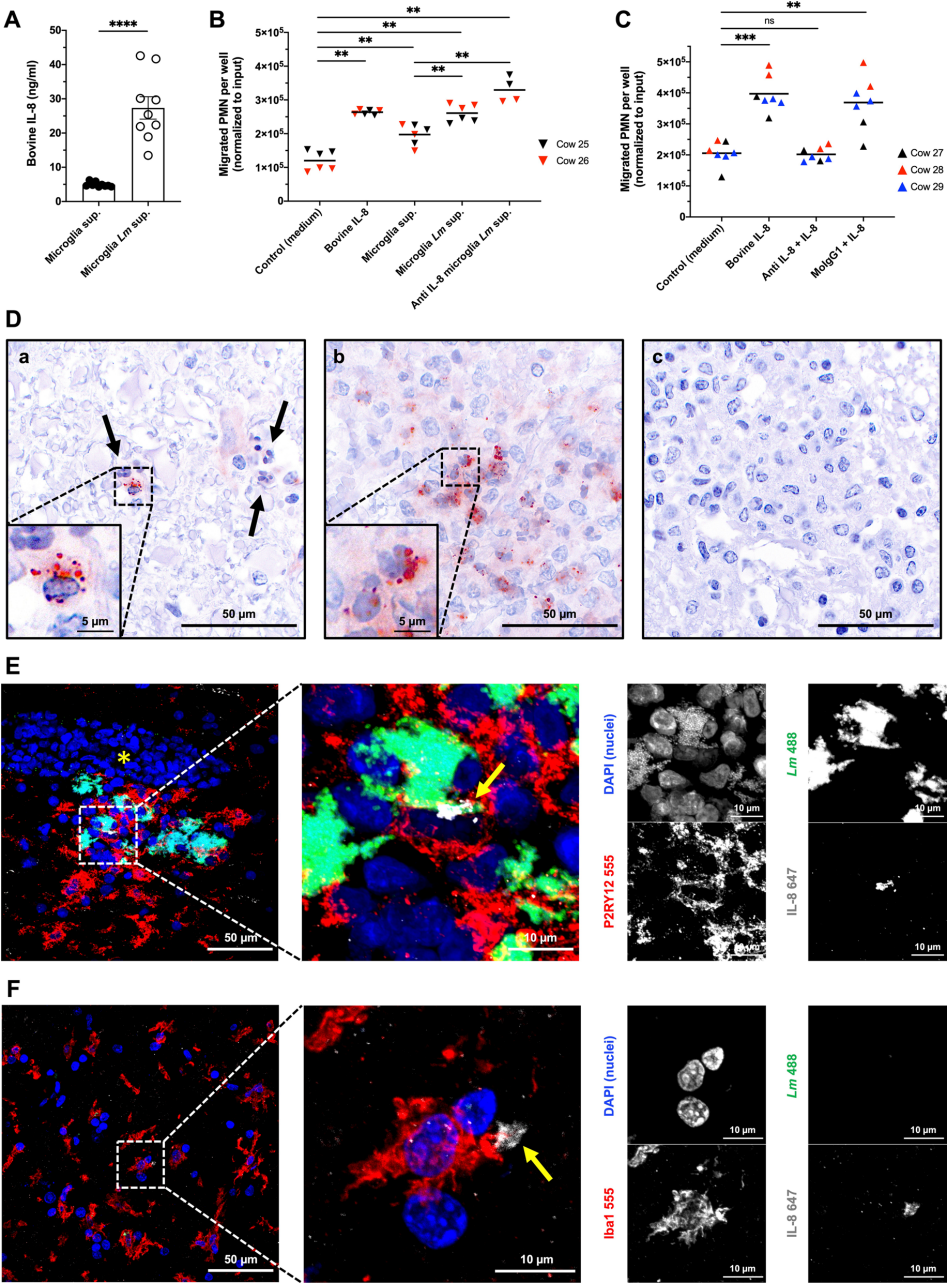
Bovine microglia express IL-8 in vitro and in situ, but IL-8 blocking in supernatant of *Lm*-infected microglia does not prevent chemotaxis of bovine PMN. **A** ELISA quantification of IL-8 from supernatants of non-infected (Microglia sup.) and *Lm*-infected (Microglia *Lm* sup.) cultured microglia. Cultured microglia exhibit a basal secretion of IL-8 into the supernatant, and infection stimulates IL-8 secretion at concentrations chemotactic for PMN. Data are represented as mean ± SEM (standard error of mean) of 3 independent experiments performed in triplicates. Statistical analysis: Mann–Whitney *U* tests (****P < 0.0001). **B** Treatment of *Lm*-infected microglial supernatant with a monoclonal anti-IL-8 antibody (anti-IL-8 microglia *Lm* sup.) does not block chemotaxis toward conditioned medium from infected microglia. Data are represented as means on a superimposed scatter dot plot of 2 independent experiments performed in duplicates (anti-IL-8 microglia *Lm* sup.) or triplicates (all other conditions). Statistical analyses: Mann–Whitney *U* tests (**P < 0.01). **C** Bovine IL-8 chemotactic activity is specifically neutralized following incubation with a monoclonal anti-IL-8 antibody (Anti IL-8 + IL-8), while IL-8 chemotactic activity is not abrogated after incubation with mouse IgG1 (MoIgG1 + IL-8). Data are represented as means on a superimposed scatter dot plot of 3 independent experiments performed in duplicates or triplicates. Statistical analyses: Mann–Whitney *U* tests (**P < 0.01; ***P < 0.001; *ns* non-significant). **D** In situ immunohistochemistry for IL-8 expression in microabscesses of cows with neurolisteriosis. **a** Early infection site, not yet developed into a microabscess, cervical spinal cord. IL-8 is expressed by a mononuclear cell morphologically compatible with microglia (inset). Notice how early recruited PMN, recognizable by their multilobed nuclei (arrows), do not yet express IL-8 at this stage. **b** Acute microabscess, cervical spinal cord. Predominant IL-8 expression by PMN gathering at the microabscess center (inset). **c** Chronic microabscess, midbrain. Mononuclear phagocytes (likely infiltrating monocyte-derived macrophages) have replaced PMN and lack IL-8 expression. **E**, **F** In situ IF for IL-8 expression in cows with neurolisteriosis. **E** Acute microabscess in the thalamus, mostly composed of microglia (P2RY12-positive cells, red) interspersed with *Lm* colonies (green), in the proximity of a perivascular cuff (yellow asterisk). Expression of IL-8 (white) by one microglia containing intracellular *Lm* (yellow arrow) is visible on higher magnification. **F** Periphery of an acute microabscess in the medulla oblongata, prevalently composed of ramified Iba1+ cells morphologically compatible with microglia (red). IL-8 expression (white) by one of such cells (yellow arrow) can be seen on higher magnification

To identify potential chemotactic candidates of microglial origin involved in bovine PMN chemotaxis, we screened transcriptomic data of genes expressed in non-infected cultured bovine microglia from our previous study [42] and identified CXCL2, CXCL5 and CXCL8 (IL-8), which are all reported to induce chemotaxis of human PMN [64], as highly expressed genes (RNA exp > 1000). We chose to focus on IL-8 for further exploration as: (I) we observed similarly high chemotaxis rates between supernatants from infected microglia and our positive control for PMN chemotaxis (bovine IL-8), (II) IL-8 has been implicated as the primary PMN chemoattractant in a wide variety of inflammatory diseases in cattle and is the best characterized CXCL chemokine in bovines [65, 66], and (III) IL-8 has been reported to functionally act as a primary PMN chemoattractant [67]. To quantify IL-8 expression in the supernatant of infected microglia, an indirect ELISA for bovine IL-8 was performed. As shown in Fig. 4A, a significantly higher concentration of IL-8 was recovered from supernatants of *Lm*-infected microglia in comparison with that of non-infected microglia. In line with our preliminary assays, indicating that synthetic bovine IL-8 elicits bovine PMN chemotaxis at concentrations between 25 and 50 ng/ml (Additional file 4: Fig. S4), our results indicate that cultured bovine microglia secrete IL-8 upon *Lm* infection at concentrations sufficient for inducing bovine PMN chemotaxis.

To assess whether PMN chemotaxis was exclusively dependent on microglial IL-8, we pre-incubated supernatants from *Lm*-infected microglia with an anti-IL-8 monoclonal antibody (Fig. 4B) which efficiently blocked bovine PMN chemotaxis towards recombinant bovine IL-8 (Fig. 4C). As illustrated in Fig. 4B, PMN chemotaxis towards treated supernatants could not be abrogated, indicating that PMN chemotaxis towards *Lm*-infected microglia supernatant was not dependent on IL-8 and suggesting that *Lm*-infected microglia secrete additional factors chemotactic for bovine PMN that are redundant to IL-8 and, hence, compensate for the lack of IL-8. Supernatant from non-infected microglia contained up to 6.2 ng/ml IL-8 (Fig. 4A) potentially explaining its modest chemotactic effect on bovine PMN (Fig. 3C).

To assess whether IL-8 was also expressed in vivo by microglia within neurolisteriosis lesions, and to corroborate our ex vivo results, we performed in situ IHC and IF on selected brain sections of cattle with neurolisteriosis. Mononuclear cells immunohistochemically positive for IL-8 and morphologically consistent with microglia were rarely observed in very early lesions (Fig. 4D, a). On the other hand, predominant IL-8 immunoreactivity was frequently observed in PMN recruited to acute/subacute microabscesses (Fig. 4D, b and Additional file 5: Fig. S5), while no IL-8-positive cells were detected in chronic microabscesses (Fig. 4D, c). To confirm that IL-8 expressing cells observed in early lesions were indeed microglia, microglia in acute microabscesses were stained in IF with antibodies against the microglial specific purinergic receptor P2RY12, *Lm* and IL-8 (Fig. 4E). In addition, we identified microglia via Iba1 expression (Fig. 4F) coupled with the lack of positivity for the monocyte/macrophage marker F13A1, as previously described [42]. IL-8 expression in microglia was confirmed in two of five cases, where very early microabscesses exclusively consisted of accumulated microglia containing intracellular bacteria without PMN and macrophages (Fig. 4E), indicating that IL-8 is expressed by infected microglia during very early neurolisteriosis lesions in vivo. Moreover, IL-8 expression was observed in uninfected Iba1+ cells morphologically compatible with microglia at the periphery of acute microabscesses (Fig. 4F), suggesting that activated microglia also trigger PMN recruitment during microabscess formation.

Overall, our data suggest that *Lm*-infected microglia promote PMN recruitment to infectious foci in the very early phase through the production of chemotactic factors, including IL-8. However, the lack of effective IL-8 blocking suggests that IL-8 is not an exclusive PMN chemoattractant and that microglia produce additional chemotactic factors. Nevertheless, IL-8 appears to be involved in microabscess formation, as it is expressed by microglia in very early lesions and is later amplified by recruited PMN themselves.

## Discussion

Although it has long been recognized that *Lm* causes marked intracerebral PMN influx in fatal meningoencephalitis of humans and ruminants [6, 68], the intracerebral immune response elicited by *Lm* remains poorly known [69, 70]. This is particularly true for PMN, as only one study investigated the role of PMN during *Lm* intrathecal infection in mice, claiming a role for PMN in bacterial containment as well as in recruitment of macrophages and lymphocytes [12]. Studies in ruminants, however, showed high *Lm* loads in PMN-rich microabscesses closely associated with neural damage, suggesting that PMN might promote neural tissue destruction in neurolisteriosis [8, 9, 71]. Evidence of PMN-associated collateral CNS damage is growing [27, 28] and dynamics of PMN-*Lm* interactions in the context of neuroinfection are poorly known. Therefore, it is essential to investigate PMN recruitment to brain lesions in neurolisteriosis to unravel mechanisms of PMN-driven anti-listerial defense and neuroimmunopathogenetic mechanisms. Knowledge of PMN recruitment will facilitate the identification of suitable therapeutic targets to counteract detrimental PMN recruitment into the delicate CNS environment.

**Fig. 5:**
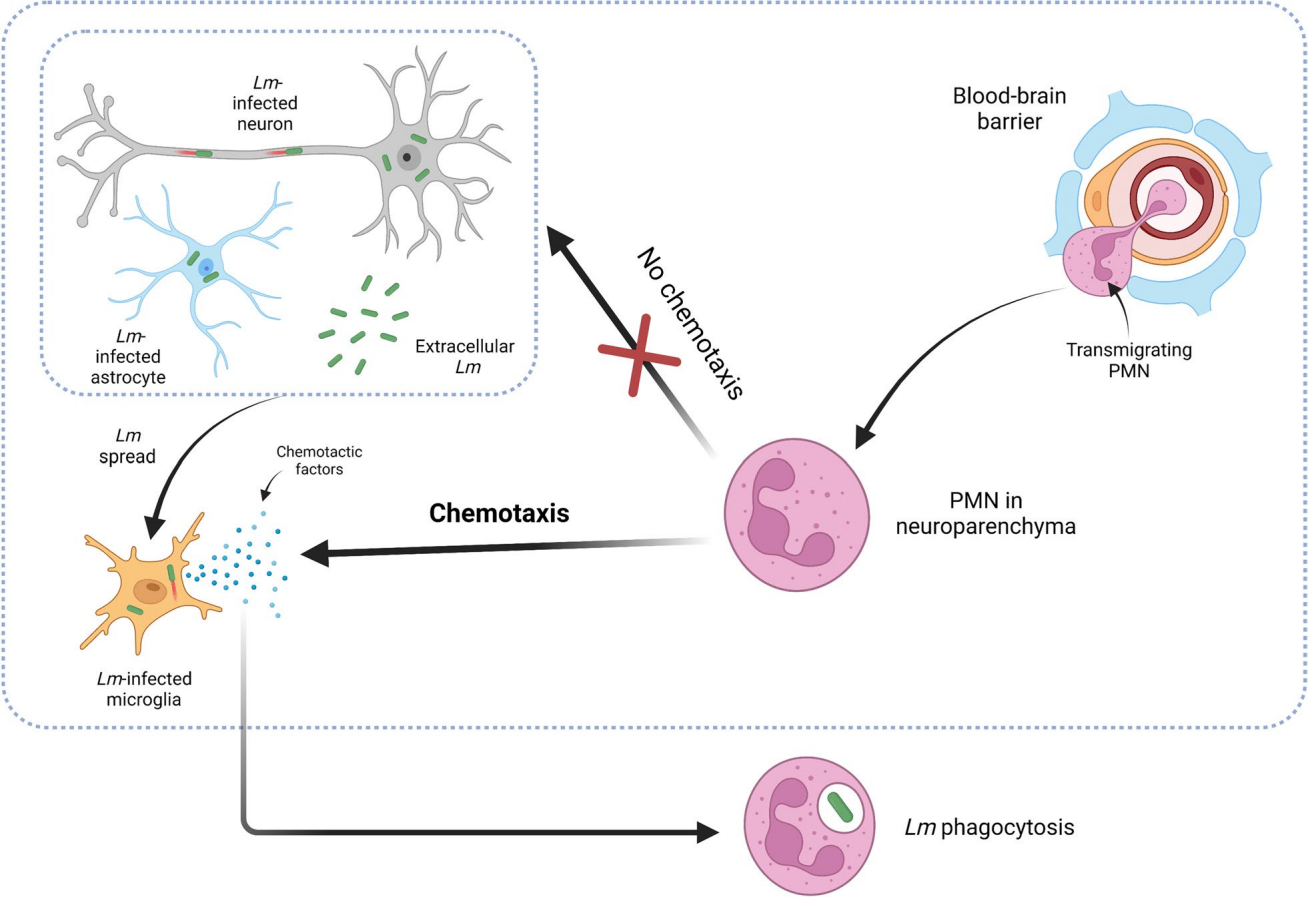
Model for PMN chemotaxis to infectious CNS foci during neurolisteriosis (large dotted box). *Lm* gains access to the CNS and infects neural cells (neurons, astrocytes, microglia) or resides extracellularly in the neuroparenchyma. PMN that entered the CNS compartment following transmigration across the BBB are unresponsive to extracellular bacteria or infected neurons and astrocytes (small dotted box), but are recruited to infectious foci by chemotactic factors of infected or activated microglia. Attracted PMN likely phagocytose *Lm* on site. As the latter step was not investigated in this study, it is not included in the large box. Created with BioRender.com

To expand our understanding of mechanisms driving PMN recruitment to CNS infectious foci during neurolisteriosis, we employed ex vivo chemotaxis assays to identify factors that contribute to chemotaxis of bovine PMN in rhombencephalitis. To this end, we compared bovine PMN chemotaxis rates toward *Lm* and endogenous brain cell-derived factors to PMN random migration. Our data provide ex vivo evidence for a lack of bovine PMN chemotaxis toward *Lm*-derived stimuli, as well as toward factors secreted by infected astrocytes and neuronal cells. In contrast, we identified microglia as major ex vivo initiators of PMN recruitment and as sources of IL-8 production upon infection with *Lm* both in vitro and in situ (Fig. 5).

To our surprise, neither *Lm* nor formyl peptides from either *E. coli* or *Lm* were chemotactic for bovine PMN, in stark contrast with widely reported results in other species (e.g., mice, human) indicating that formyl peptides are strong chemoattractants for PMN (reviewed in [72]). PMN chemotactic responses toward *Lm* remain largely unclear but have been (at least partially) attributed to a direct chemotactic effect of bacterial components on PMN including formyl peptides [73]. In vitro studies assessing murine PMN chemotaxis toward *Lm* formyl peptides, however, provided contradicting results, reporting either a positive chemotactic effect [36, 37] or lack thereof [58]. PMN recruitment to infection sites in vivo was reported to be dependent on formyl peptide-receptors (FPRs) and attributed to recognition of *Lm* formyl peptides [36], although the potential chemotactic contribution of formyl peptides released from damaged host cells was not excluded in this study. While data on the effect of *Lm*-derived formyl peptides on bovine PMN chemotaxis is lacking, available data on the chemotactic effect of *E. coli*-derived fMLP toward bovine PMN is contradicting. Some authors reported a chemotactic effect of fMLP [53–55], while others, in line with our results, failed to identify a bovine PMN migratory response toward bacterial peptides (formylated and non-formylated), bacterial culture supernatants, and viable or sonicated Gram-positive and Gram-negative bacteria [50–52]. The latter studies and our findings are supported by the reported lack of FPRs on bovine PMNs [52]. In addition, PMN from ungulates and carnivores are unresponsive toward *E. coli*-derived fMLP and bacterial culture filtrates from numerous bacterial species (reviewed in [72]), highlighting relevant species-specific differences in PMN responsiveness to chemotactic stimuli.

Similarly, *Lm* supernatants and whole *Lm*, whether heat-killed or viable, opsonized or non-opsonized, were not able to induce chemotaxis of bovine PMN. Human PMN have been shown to be incapable of migrating toward *Lm* cell wall fractions unless PMN migration was allowed to occur toward fresh serum containing such fractions, leading to complement activation [74]. Further studies are needed to clearly identify factors and mechanisms responsible for PMN chemotaxis toward *Lm*, especially in non-murine species. However, data reported in human PMN [74] are in line with our results in bovine PMN showing no direct chemotactic response to *Lm* or *Lm*-derived factors. These observations suggest that host-derived factors rather than bacterial components per-se are primarily responsible for PMN chemotaxis toward *Lm* infectious foci in both cattle and humans. In this context, the lack of bovine PMN chemotaxis toward opsonized *Lm* might be indicative of the requirement of soluble, rather than bacteria-fixed, complement components for PMN chemotaxis.

The contribution of endogenous brain cells to PMN recruitment during neuroinfection remains only partially understood. PMN chemotaxis appears to be primarily dependent on factors secreted by microglia and astrocytes rather than neurons. Indeed, previous work demonstrated that PMN-attracting chemokines (e.g., CXCL1, CXCL2, IL-8) were produced by murine microglia in a *S. aureus* brain abscess model [75] and by human astrocytes infected with *B. burgdorferi* and Group B *Streptococcus* [29, 76], as well as by human microglia [77] and murine astrocytes [78] upon LPS stimulation, while murine primary neurons exposed to LPS displayed expression and release of CXCL1 at much lower levels than mixed glial cultures [33]. Similarly, our data show that in the bovine ex vivo chemotaxis model microglia are responsible for the production of PMN chemoattractants upon *Lm* infection and that *Lm*-infected microglia secrete chemotactic levels of IL-8 in vitro and in situ, suggesting that microglia may orchestrate PMN recruitment during neurolisteriosis. This view is supported by previous observations indicating that microglia are preferential targets of *Lm* in both dissociated cultures and organotypic brain slices of rodents and ruminants [79–82], and that *Lm*-infected murine microglia upregulate CXCL2 in an *actA*-dependent manner [83]. Despite the secretion of IL-8 at concentrations capable of eliciting PMN chemotaxis, treatment of microglia supernatant with a blocking anti-IL-8 antibody surprisingly did not prevent chemotaxis. Our data suggest that IL-8 is not the main chemotactic factor responsible for PMN chemotaxis and that infected microglia produce additional chemoattractive factors that compensate for the lack of IL-8. These results are in line with previous observations indicating that CXCL chemokines may show redundant chemotactic effects at inflammatory sites, including in the CNS [84–86]. The identification of such additional factors released by bovine microglia upon *Lm* infection is currently ongoing. Indeed, a recent transcriptomic study indicates that bovine microglia upregulate several chemokines including CXCL2, CXCL3, CXCL5, and IL-8 (all known chemoattractants for human PMN, reviewed in [64]) upon culture [42]. The upregulation of chemokine expression in non-infected microglia is attributable to activation caused by experimental culture procedures and is likely to be responsible for the modest PMN chemotaxis that was observed toward supernatants of non-infected microglia in our study. Our observation that IL-8 is expressed by microglia located at the periphery of established microabscesses supports the view that activation is sufficient to trigger chemokine production by microglia. In addition to CXCL chemokines, leukotriene B_4_ (LTB4)_,_ previously shown to promote PMN infiltration into the damaged brain [87] and to be chemoattractactive for bovine PMN [88, 89], may be involved in PMN recruitment by microglia, even though the above mentioned transcriptomic study has not identified LTB4 upregulation in cultured microglia [42]. In summary, our data suggest a partial but not exclusive role for microglia-derived IL-8 in PMN recruitment and microabscess formation during early neurolisteriosis.

While the lack of PMN chemotaxis toward the supernatant of infected FBBC-1 cells is in line with previous observations indicating a marginal role for neurons in secreting significant quantities of PMN chemoattractants [33], the absence of enhanced chemotaxis toward infected astrocyte medium was unexpected, given the production of PMN-specific chemoattractants, including IL-8, reported in previous studies [29, 76, 78]. This discrepancy might be explained by the different animal and/or bacterial species used in our study in comparison with these previous works [29, 76, 78], or by differences in the MOI used (= 10 in our experiments, vs ≥ 40 used in previously mentioned studies to determine chemokine transcripts). Moreover, our in vitro culture system might not fully recapitulate in vivo properties of bovine astrocytes due to alterations in their phenotypic profile caused by culture conditions, as previously described in other species (reviewed in [90, 91]). In addition, as the interplay between chemokine axes of various neural cells was shown to promote PMN recruitment during neuroinfection in vivo [92], we cannot exclude that neural cells other than microglia may also contribute to PMN chemotaxis during neurolisteriosis in vivo. Despite this, we also did not observe any IL-8 expression in cells morphologically identifiable as astrocytes in situ, suggesting that, in bovines, astrocytes do not secrete detectable quantities of IL-8 upon *Lm* infection.

This study provides valuable insights into mechanisms of PMN chemotaxis in neurolisteriosis, identifying a primary role for microglia in initiating bovine PMN chemotaxis to infectious foci in neurolisteriosis. The evaluation of chemotactic effects in this simplified PMN chemotaxis model primarily explains local PMN chemotaxis to infectious foci once inside the neuroparenchyma but not the recruitment of PMN into the brain. The dissection of the latter requires a model mimicking the PMN extravasation cascade under physiological flow across the BBB. Nevertheless, the lack of a direct chemotactic effect of *Lm* and the requirement of microglia-derived chemotactic factors to recruit PMNs to sites of *Lm* infection suggests that *Lm* may delay PMN response in the brain through avoidance of detection by microglia, the sentinels in the CNS. In vivo observations of *Lm* within axons of mice and ruminants during early stages of encephalitis without infiltrations of inflammatory cells, i.e., PMN [8, 93], support this view and suggest that *Lm* could exploit this PMN evasion strategy to facilitate silent intracerebral spread.

## Conclusions

Overall, our study indicates that host-derived factors are key components in initiating bovine PMN chemotaxis to infectious foci in neurolisteriosis. We demonstrate that microglia-derived factors are responsible for eliciting bovine PMN chemotaxis, while neuronal cells and astrocytes do not appear to participate in PMN recruitment upon infection. Microglia secrete IL-8 upon *Lm* infection both in vitro and in situ suggesting that IL-8 contributes to PMN chemotaxis into infection foci. However, the lack of a blocking effect of anti-IL8 antibody indicates that IL-8 is not the exclusive chemotactic factor released by bovine microglia and that microglia produce additional potent chemotactic factors contributing to PMN chemotaxis. We also show that *Lm* and associated bacterial factors, including formyl peptides, are inefficient in eliciting chemotaxis of bovine PMN unlike in other species and, hence, have identified a second species-specific interaction of *Lm* with host cells additionally to the species-specific binding of the bacterial surface protein Internalins A and B to their respective host receptors [94, 95]. Although its impact on species susceptibility and evolutionary adaptation to *Lm* remains to be shown, this finding underscores the importance of considering species-specific differences in PMN functions when performing comparative studies on innate immunity in listeriosis.

## Supplementary Information

**Additional file 1: Figure S1. **Establishment of gating strategy for FACS analysis of migrated bovine PMN. Preliminary gating in staining controls (**A-C**) and application of these gating strategies on input samples, as shown in a representative input sample (**D**). Represented plots were obtained from the same animal. **A **Gating of unstained cells to exclude contaminants, cell debris and cell aggregates. Unstained cells are gated for PMN based on the FSC-A vs SSC-A plot (PMN gating), then singlet PMN are selected based on an FSC-A vs FSC-H plot (singlet gating). The same PMN gating and singlet gating settings (with the exception of singlet gating for **B**, see below) is consistently applied to all other controls and analyzed samples in the same experiment. **B **Gating of viable and dead cells to exclude dead cells. Control sample for viability gating is obtained by mixing untreated cells with cell death marker (LD) stained dead cells (i.e., cells incubated for 4 min at 99°C then cooled down on ice for 5 min) at a ratio of 1/1. PMN gating is applied as in **A**, while singlet gating is adjusted to include both dead and alive PMN. Dead PMN are then separated from alive PMN based on their positivity for LD. **C** Gating of CellTracker Green (CTG)- positive and negative cells to exclude CTG-negative cells. Control sample for CTG staining is obtained by mixing unstained cells with cells stained for CTG at a 1/1 ratio. PMN gating and singlet gating are sequentially applied as in **A**, then single PMN are gated for their CTG positivity or lack thereof (CTG gating). **D **Representative gating strategy of an input sample. PMN and singlets are gated as in **A **to exclude contaminants, cell debris and aggregates, then viability gating is applied as in **B **to exclude dead cells. Single viable PMN are then gated for CTG to exclude CTG-negative PMN. The same gating strategy adopted for the input is also applied to all migrated cells in all different conditions tested.

**Additional file 2: Figure S2. **Gating of CH138A-positive bovine granulocytes to confirm the gating strategy shown in Additional file 1: Fig. S1. **A **Cells stained with the anti-granulocyte antibody CH138A are gated for CH138A-positive cells (granulocytes) and CH138A-negative cells (contaminants) based on the unstained negative control (not shown). The overlay figure shows the localization of bovine granulocytes (light blue) and contaminant cells (red) on the FSC-A vs SSC-A plots. **B **Overlay plot of gated PMN from Additional file 1: Fig. S1D (light blue) against the overall cell population (red) shows that gated PMN are located in the same FSC-A vs SSC-A area as CH138A+ granulocytes gated according to Additional file 1: Fig. S2A, confirming that the population gated using the strategy illustrated in Additional file 1: Fig. S1D is prevalently constituted by PMN.

**Additional file 3: Figure S3. **Immunophenotypization of primary bovine astrocytes. **A** Representative immunofluorescent staining of cultured primary bovine astrocytes (1 day post-seeding). Cultured cells show moderate to strong granular-fibrillary cytoplasmic GFAP positivity (**a**) and marked fibrillary cytoplasmic positivity for Vimentin (**b**), while only few cells display granular cytoplasmic positivity for S100 (**c**). Iba1-positive cells constitute contaminating microglia (**d**), while no cells stain with Olig2 (**e)** and NeuN (**f**), indicating the absence of contaminating neurons and oligodendrocytes, respectively. Rabbit Ig fraction (RbIg, **g**) and mouse IgG1 (MoIgG1, **h**) were used as negative controls. Nuclei are stained blue with DAPI. 60x magnification. **B **Percentage of immunolabelled cells per field of view (FOV) in 17 independent FOV. > 95% of cells are GFAP+ and Vimentin+, indicating high astrocyte purity, while < 1% of cells express S100 and 1-2% are contaminating microglia (Iba1+ cells). Data are expressed as mean ± SEM.

**Additional file 4: Figure S4. **Recombinant bovine IL-8 (IL-8) elicits optimal chemotaxis of bovine PMN at concentrations between 25 and 50 ng/ml, while the chemotactic effect drops at 100ng/ml. Data are represented as means on a superimposed scatter dot plot of 1 to 3 independent experiments, each performed in triplicates.

**Additional file 5**: **Figure** **S5.** IL-8 production by PMN in an acute microabscess in the medulla oblongata of a cow. The microabscess core is mainly composed of Iba1-negative cells with polymorphonuclear nuclei (compatible with PMN), while Iba1+ phagocytes (red) are located peripherally. IL-8 immunoreactivity (white) localizes predominantly to PMN in the center. At higher magnification of the periphery, IL-8 is expressed by PMN (full yellow arrow) and Iba1+ cells (dotted yellow arrow). Nuclei are stained blue with DAPI.

**Additional file 6: Methods S1. **Generation of Δ*hly-Lm *deletion mutant.

**Additional file 7: Methods S2. **CH138A staining of bovine PMN for FACS analysis.

**Additional file 8: Table S1. **Clinical data of donor cows.

**Additional file 9: Table S2. **Methods and antibodies used for astrocyte immunofluorescence.

**Additional file 10: Table S3. **Conditions tested in the Transwell chemotaxis assay.

**Additional file 11: Table S4. **Analyzed datasets.

## Data Availability

The data sets analyzed during the current study are included within the article as Additional file 11: Table S4.

## Abbreviations

BBB: Blood–brain barrier
bFGF: Basic fibroblast growth factor
BHI: Brain–heart infusion
BSA: Bovine serum albumin
CAM: Chemotaxis assay medium
CDC: Cholesterol-dependent cytolysin
CFU: Colony-forming unit
CNS: Central nervous system
CTG: CellTracker Green CMFDA
DAPI: 4′,6-Diamidino-2-phenylindole
DMEM: Dulbecco’s modified Eagle’s medium
EDTA: Ethylenediaminetetraacetic acid
ELISA: Enzyme-linked immunosorbent assay
FACS: Fluorescence-activated cell sorting
FBBC-1: Fetal bovine brain cells-1
FCS: Fetal calf serum
FFPE: Formalin-fixed paraffin-embedded
fMIVIL: N-formyl-MIVIL
fMIVTLF: N-formyl-MIVTLF
fMLP: N-formyl-Met-Leu-Phe
FOV: Field of vie
FPRs: Formyl peptide-receptors
FSC: Forward scatter
GFAP: Glial fibrillary acidic protein
HK-*Lm*: Heat-killed WT-*Lm*
Iba1: Ionized calcium binding adaptor molecule 1
IF: Immunofluorescence
IHC: Immunohistochemistry
IL-8: Interleukin-8 (alias: CXCL8)
LD: LIVE/DEAD fixable near-IR dead cell stain
LLO: Listeriolysin O
*Lm*: *Listeria monocytogenes*
LPS: Lipopolysaccharide
LTB4: Leukotriene B_4_
MOI: Multiplicity of infection
MoIgG1: Mouse IgG1 isotype control
NGS: Normal goat serum
OD: Optical density
Olig2: Oligodendrocyte transcription factor 2
PAMPs: Pathogen-associated molecular patterns
PBS-T: PBS-Tween
PBS: Phosphate-buffered saline
PFA: Paraformaldehyde
PMN: Polymorphonuclear neutrophils
PRRs: Pattern-recognition receptors
RbIg: Rabbit Ig fraction
RT: Room temperature
SSC: Side scatter
WT-*Lm*: Wild-type *Listeria monocytogenes*
