# Supplementary figures and images for: Bovine neutrophil chemotaxis to Listeria monocytogenes in neurolisteriosis depends on microglia-released rather than bacterial factors
Source: J Neuroinflammation. 2022 Dec 16;19:304. doi: 10.1186/s12974-022-02653-1 (PMC9758797; doi:10.1186/s12974-022-02653-1)

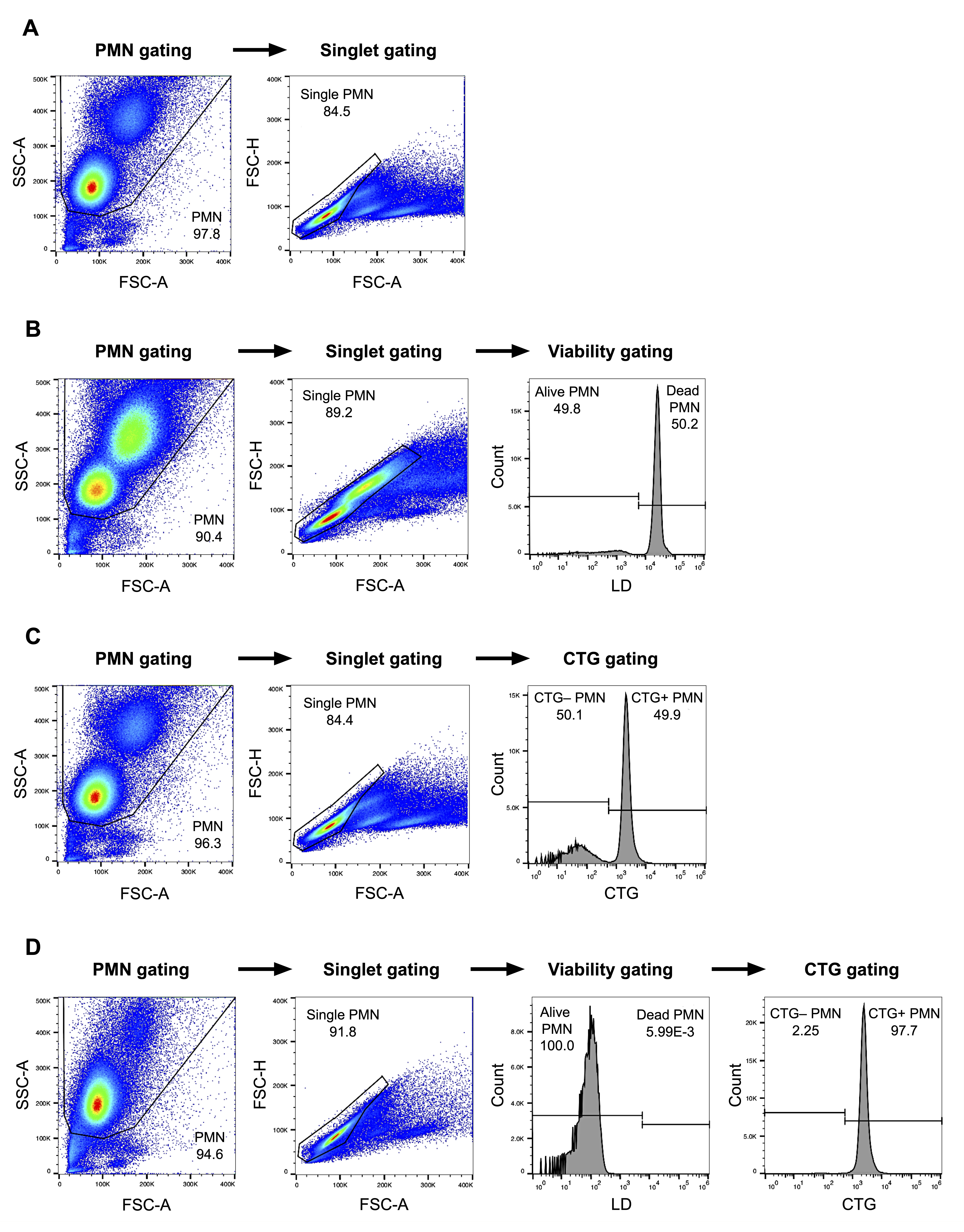

Supplement: Supplementary file 1 — Additional file 1: Figure S1. Establishment of gating strategy for FACS analysis of migrated bovine PMN. Preliminary gating in staining controls (A-C) and application of these gating strategies on input samples, as shown in a representative input sample (D). Represented plots were obtained from the same animal. A Gating of unstained cells to exclude contaminants, cell debris and cell aggregates. Unstained cells are gated for PMN based on the FSC-A vs SSC-A plot (PMN gating), then singlet PMN are selected based on an FSC-A vs FSC-H plot (singlet gating). The same PMN gating and singlet gating settings (with the exception of singlet gating for B, see below) is consistently applied to all other controls and analyzed samples in the same experiment. B Gating of viable and dead cells to exclude dead cells. Control sample for viability gating is obtained by mixing untreated cells with cell death marker (LD) stained dead cells (i.e., cells incubated for 4 min at 99°C then cooled down on ice for 5 min) at a ratio of 1/1. PMN gating is applied as in A, while singlet gating is adjusted to include both dead and alive PMN. Dead PMN are then separated from alive PMN based on their positivity for LD. C Gating of CellTracker Green (CTG)- positive and negative cells to exclude CTG-negative cells. Control sample for CTG staining is obtained by mixing unstained cells with cells stained for CTG at a 1/1 ratio. PMN gating and singlet gating are sequentially applied as in A, then single PMN are gated for their CTG positivity or lack thereof (CTG gating). D Representative gating strategy of an input sample. PMN and singlets are gated as in A to exclude contaminants, cell debris and aggregates, then viability gating is applied as in B to exclude dead cells. Single viable PMN are then gated for CTG to exclude CTG-negative PMN. The same gating strategy adopted for the input is also applied to all migrated cells in all different conditions tested. [file 12974_2022_2653_MOESM1_ESM.jpg]

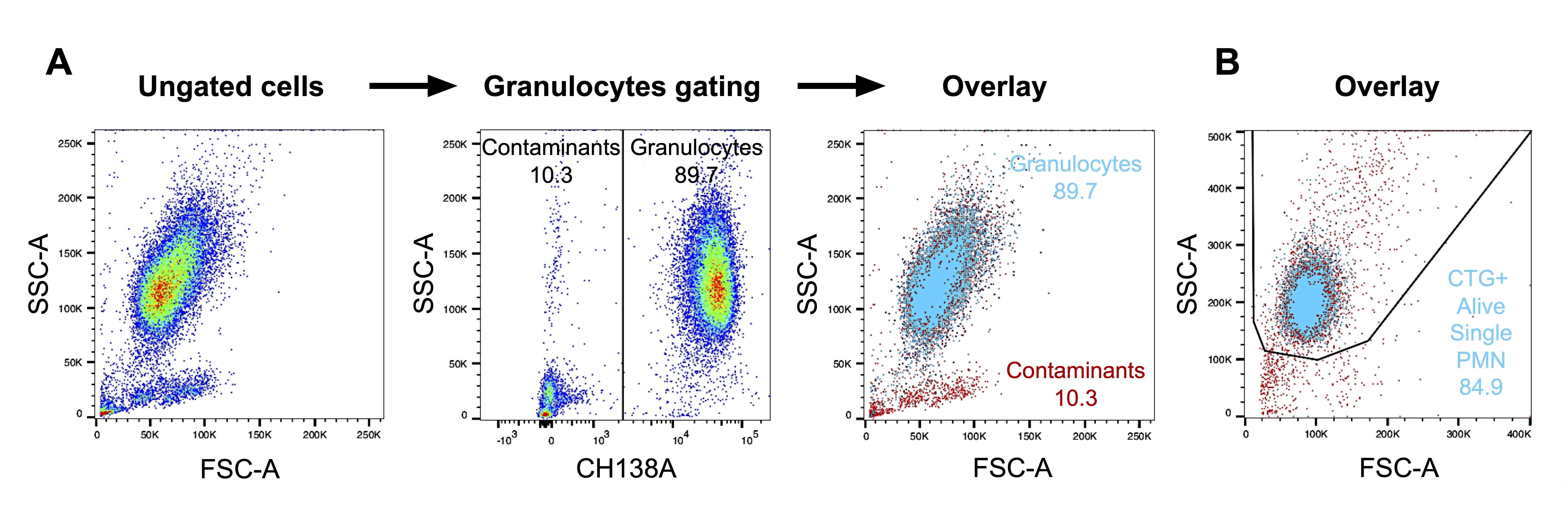

Supplement: Supplementary file 2 — Additional file 2: Figure S2. Gating of CH138A-positive bovine granulocytes to confirm the gating strategy shown in Additional file 1: Fig. S1. A Cells stained with the anti-granulocyte antibody CH138A are gated for CH138A-positive cells (granulocytes) and CH138A-negative cells (contaminants) based on the unstained negative control (not shown). The overlay figure shows the localization of bovine granulocytes (light blue) and contaminant cells (red) on the FSC-A vs SSC-A plots. B Overlay plot of gated PMN from Additional file 1: Fig. S1D (light blue) against the overall cell population (red) shows that gated PMN are located in the same FSC-A vs SSC-A area as CH138A+ granulocytes gated according to Additional file 1: Fig. S2A, confirming that the population gated using the strategy illustrated in Additional file 1: Fig. S1D is prevalently constituted by PMN. [file 12974_2022_2653_MOESM2_ESM.jpg]

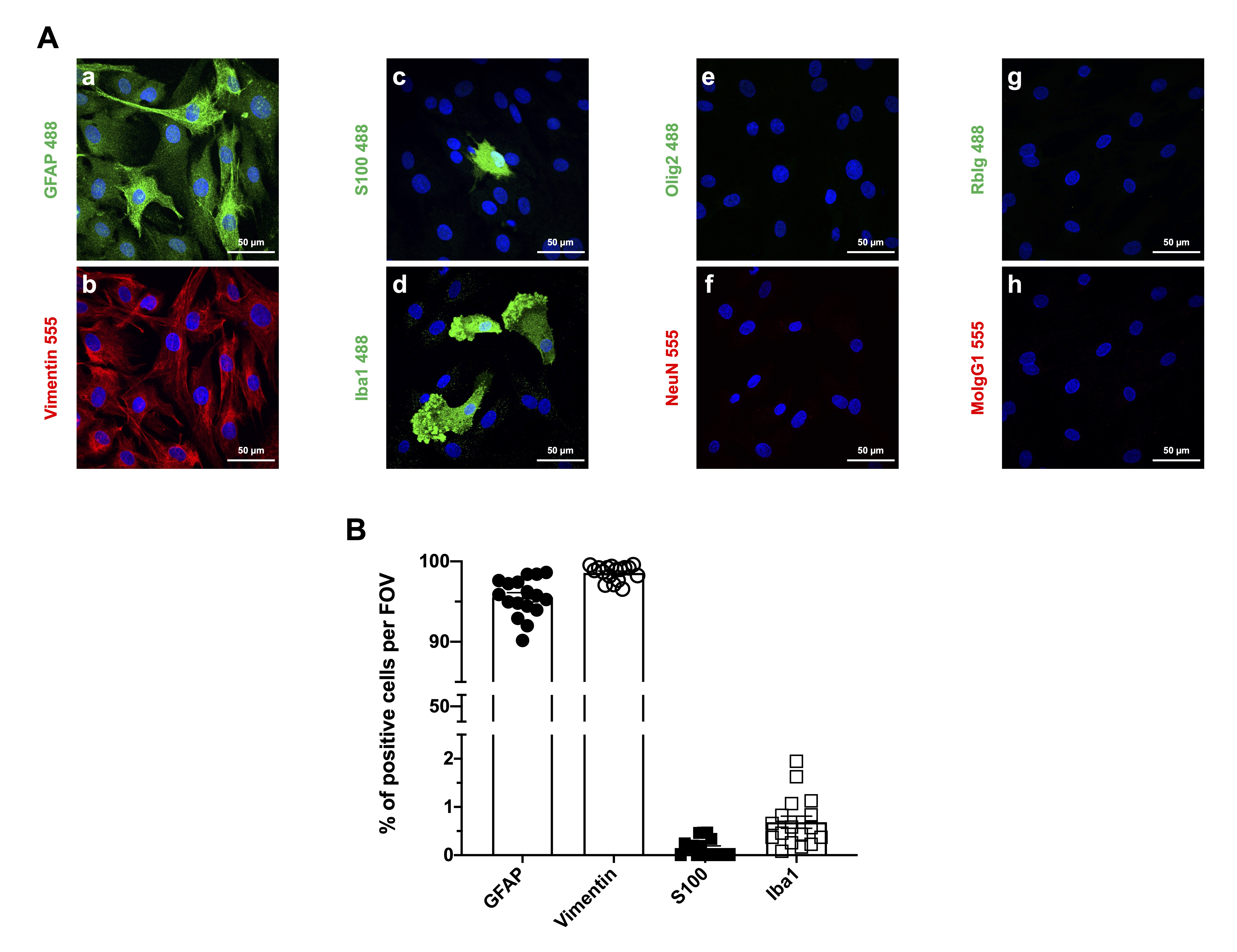

Supplement: Supplementary file 3 — Additional file 3: Figure S3. Immunophenotypization of primary bovine astrocytes. A Representative immunofluorescent staining of cultured primary bovine astrocytes (1 day post-seeding). Cultured cells show moderate to strong granular-fibrillary cytoplasmic GFAP positivity (a) and marked fibrillary cytoplasmic positivity for Vimentin (b), while only few cells display granular cytoplasmic positivity for S100 (c). Iba1-positive cells constitute contaminating microglia (d), while no cells stain with Olig2 (e) and NeuN (f), indicating the absence of contaminating neurons and oligodendrocytes, respectively. Rabbit Ig fraction (RbIg, g) and mouse IgG1 (MoIgG1, h) were used as negative controls. Nuclei are stained blue with DAPI. 60x magnification. B Percentage of immunolabelled cells per field of view (FOV) in 17 independent FOV. > 95% of cells are GFAP+ and Vimentin+, indicating high astrocyte purity, while < 1% of cells express S100 and 1-2% are contaminating microglia (Iba1+ cells). Data are expressed as mean ± SEM. [file 12974_2022_2653_MOESM3_ESM.jpg]

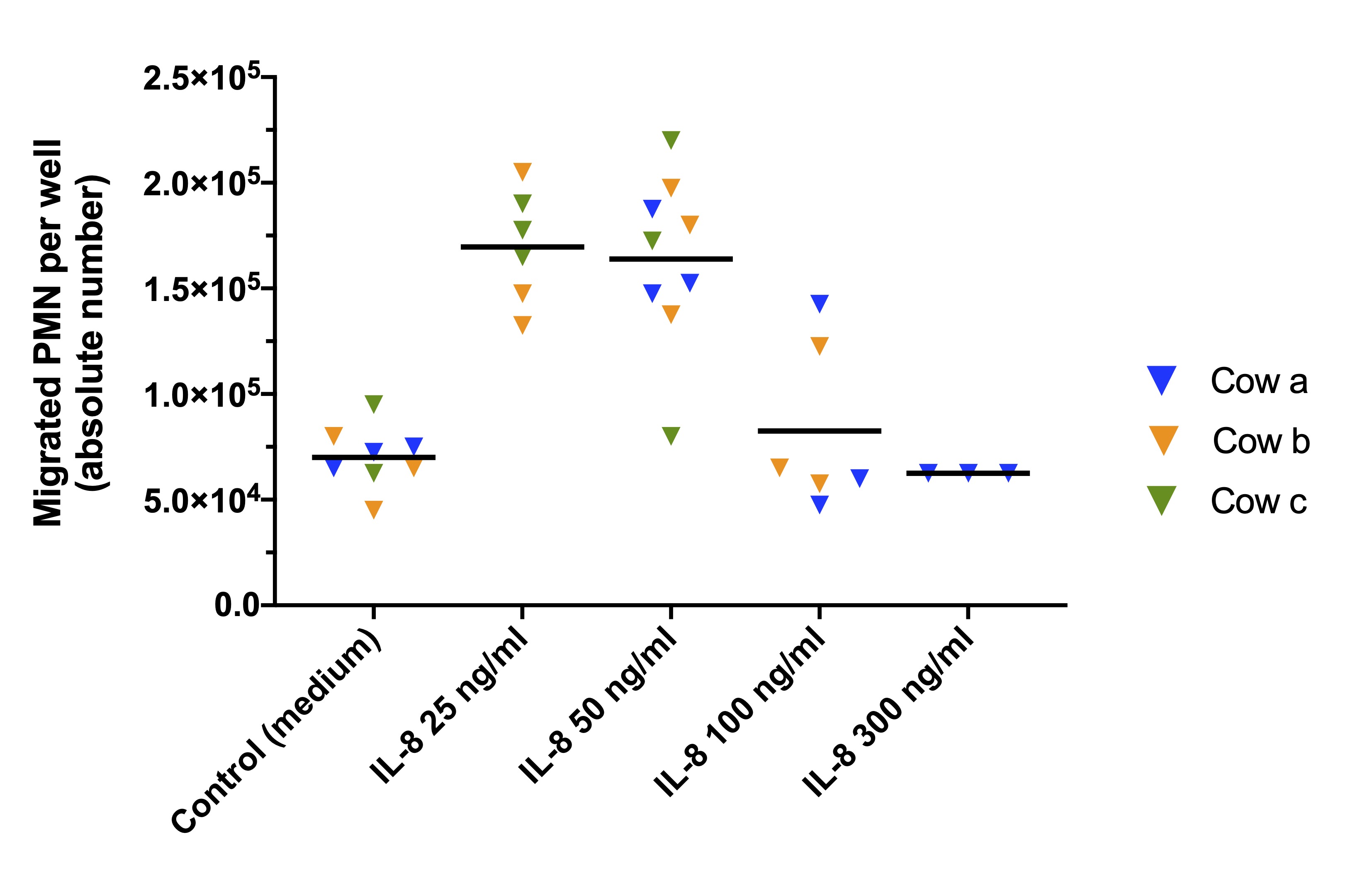

Supplement: Supplementary file 4 — Additional file 4: Figure S4. Recombinant bovine IL-8 (IL-8) elicits optimal chemotaxis of bovine PMN at concentrations between 25 and 50 ng/ml, while the chemotactic effect drops at 100ng/ml. Data are represented as means on a superimposed scatter dot plot of 1 to 3 independent experiments, each performed in triplicates. [file 12974_2022_2653_MOESM4_ESM.jpg]

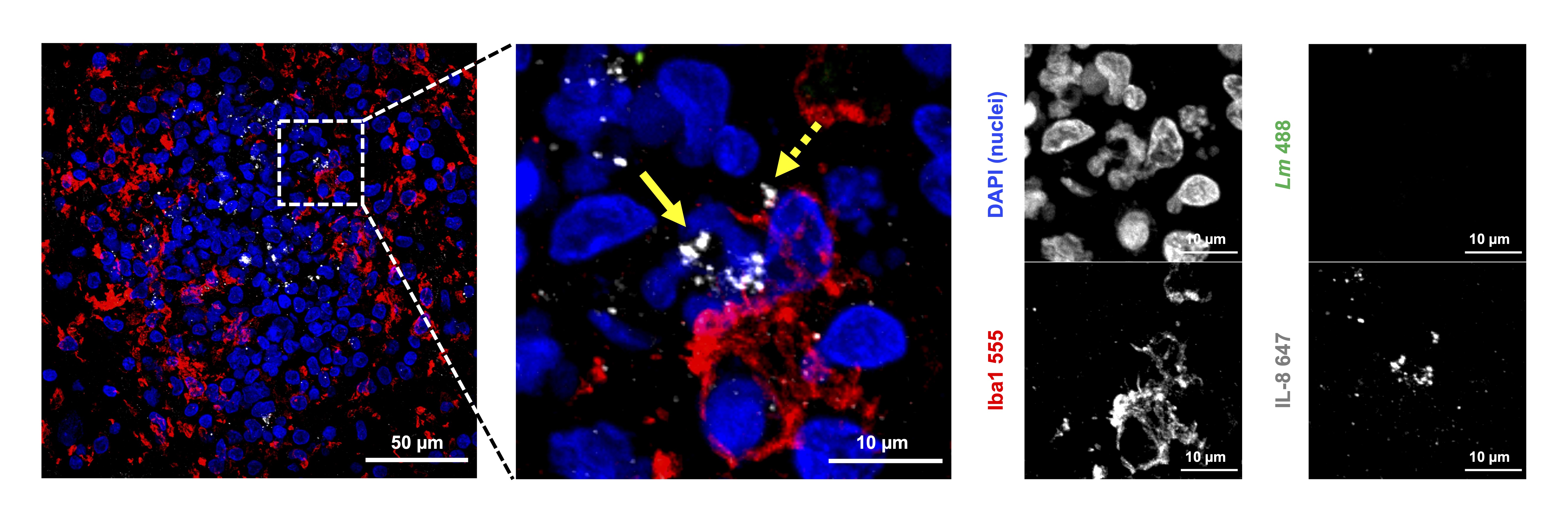

Supplement: Supplementary file 5 — Additional file 5: Figure S5. IL-8 production by PMN in an acute microabscess in the medulla oblongata of a cow. The microabscess core is mainly composed of Iba1-negative cells with polymorphonuclear nuclei (compatible with PMN), while Iba1+ phagocytes (red) are located peripherally. IL-8 immunoreactivity (white) localizes predominantly to PMN in the center. At higher magnification of the periphery, IL-8 is expressed by PMN (full yellow arrow) and Iba1+ cells (dotted yellow arrow). Nuclei are stained blue with DAPI. [file 12974_2022_2653_MOESM5_ESM.jpg]
