# Supplementary material for: Bovine neutrophil chemotaxis to Listeria monocytogenes in neurolisteriosis depends on microglia-released rather than bacterial factors
Source: J Neuroinflammation. 2022 Dec 16;19:304. doi: 10.1186/s12974-022-02653-1 (PMC9758797; doi:10.1186/s12974-022-02653-1)
Supplement: Supplementary file 6 — Additional file 6: Methods S1. Generation of Δhly-Lm deletion mutant. [file 12974_2022_2653_MOESM6_ESM.docx]

**Additional files: Additional methods 1**

**Generation of Δ*hly-Lm* deletion mutant**

The Δ*hly-Lm* was generated as an in-frame deletion mutant from the parental *Lm* strain JF5203 (lineage I, clonal complex 1, sequence type 1, https://www.ncbi.nlm.nih.gov/nuccore/NZ_LT985474.1; WT-*Lm*) using the pMAD plasmid, as previously described [1]. DNA extracted from WT-*Lm* was used as the template for the amplification of the up- and downstream regions of *hly* by PCR (Roche, Basel, Switzerland) using the primer pairs Δ*hly*_1_fw_XmaI (ATATATCCCGGGTTGCTCGTGTCAGTTCTGGG)/ Δ*hly*_2_rv (TTCGATTGGTTTCATGGGTTTCACTCTCC) and Δ*hly*_3_fw (CCCATGAAACCAATCGAATAATTTTAAAAG)/ Δ*hly*_4_rv_XmaI (ATATATGTCGACTGACCAATGGCTTCAAATGC), respectively. The two amplicons were connected by splice over extension PCR using primers Δ*hly*_1_fw_SalI and Δ*hly*_4_rv_XmaI. Deletion and selection were performed as described previously [2, 3]. The *hly* deletion of the final clone was confirmed by PCR.

**References:**

[1]: Arnaud M, Chastanet A, Débarbouillé M (2004) New vector for efficient allelic replacement in naturally nontransformable, low-GC-content, gram-positive bacteria. Appl Environ Microbiol 70:6887–6891. doi: 10.1128/AEM.70.11.6887-6891.2004.

[2]: Henke D, Rupp S, Gaschen V, Stoffel MH, Frey J, Vandevelde M, Oevermann A (2015) Listeria monocytogenes spreads within the brain by actin-based intra-axonal migration. Infect Immun 83:2409–2419. doi: 10.1128/IAI.00316-15.

[3]: Rupp S, Bärtschi M, Frey J, Oevermann A (2017) Hyperinvasiveness and increased intercellular spread of Listeria monocytogenes sequence type 1 are independent of listeriolysin S, internalin F and internalin J1. J Med Microbiol 66:1053–1062. doi: 10.1099/jmm.0.000529.
