# Supplementary material for: Bovine neutrophil chemotaxis to Listeria monocytogenes in neurolisteriosis depends on microglia-released rather than bacterial factors
Source: J Neuroinflammation. 2022 Dec 16;19:304. doi: 10.1186/s12974-022-02653-1 (PMC9758797; doi:10.1186/s12974-022-02653-1)
Supplement: Supplementary file 7 — Additional file 7: Methods S2. CH138A staining of bovine PMN for FACS analysis. [file 12974_2022_2653_MOESM7_ESM.docx]

**Additional files: additional methods 2**

**CH138A staining of bovine PMN for FACS analysis**

10^6^ freshly extracted bovine PMN were resuspended in 100 μl of ice-cold PBS and stained with the anti-bovine granulocyte monoclonal antibody CH138A (1:100; IgG1, Cat. No WS0608B-100, Kingfisher Biotech, Inc.) for 15 min on ice. Cells were washed with 1 ml of PBS and centrifuged at 350 x g for 10 min at 4°C, after which the supernatant was discarded and PMN were incubated in PBS containing a PE-conjugated goat anti-mouse monoclonal IgM (1:100; PA186003, Invitrogen) for 15 min on ice. Cells were washed with PBS and centrifuged as above, following which the supernatant was removed and PMN were resuspended in 200 μl of PBS. A sample containing unstained PMN was used as negative control. Samples were measured with a FACS Canto II flow cytometer (BD Biosciences) and data analysis was conducted using the FlowJoTM software (Tree Star, Ashland, OR, USA).
