# Supplementary material for: Bovine neutrophil chemotaxis to Listeria monocytogenes in neurolisteriosis depends on microglia-released rather than bacterial factors
Source: J Neuroinflammation. 2022 Dec 16;19:304. doi: 10.1186/s12974-022-02653-1 (PMC9758797; doi:10.1186/s12974-022-02653-1)
Supplement: Supplementary file 9 — Additional file 9: Table S2. Methods and antibodies used for astrocyte immunofluorescence. [file 12974_2022_2653_MOESM9_ESM.pdf]

Table S2 – Methods and antibodies used for astrocyte immunofluorescence

A) Fixation/<br>B) Permeabilisation/<br>C) Blocking	Primary antibody*	Species	Clone	Dilution, incubation time	Washes	Secondary antibodies/ Nuclear stain/ Incubation	Washes	Mounting
A) 15' in 4% PFA at RT<br><br>B) 5' PBS-T (0.5%)<br>5' PBS-Triton-X 100 (0.5%)<br>5' PBS-T<br><br>C) 30' PBS-T with 10% NGS	GFAP (Dako)	Rabbit	Polyclonal	1:1000, 1h at RT	3 x 5' PBS-T	Alexa Fluor 488-conjugated goat anti-rabbit IgG (Life technologies, 1:500)<br><br>Alexa Fluor 555-conjugated goat anti-mouse IgG (Life technologies, 1:500)<br><br>DAPI (Invitrogen, 1:10,000)	3 x 5' PBS-T<br><br>Rinsed in distilled water	Glycergel Mounting Medium (Dako, Glostrup, Denmark)
	S100 (Dako)	Rabbit	Polyclonal	1:600; 1h at RT				
	Vimentin (Dako)	Mouse	VIM3B4/1	1:100; 1h at RT				
	NeuN (Millipore)	Mouse	A60	1:200; 1h at RT				
	Olig2 (Millipore)	Rabbit	Polyclonal	1:200; 1h at RT				
	Iba1 (Wako)	Rabbit	Polyclonal	1:500; 1h at RT				

						1h at RT in PBS-T, protected from light		

* Primary antibodies were diluted and incubated in PBS-T with 10% NGS, unless otherwise indicated.

Rabbit Ig fraction (RbIg, final concentration 3.8 µg/ml; X0903, Dako) and Mouse IgG1 Isotype Control (MoIgG1, final concentration 50 µg/ml; MAB002, R&D systems) were used as negative controls for primary anti-rabbit and anti-mouse antibodies, respectively.
