# Supplementary material for: Bovine neutrophil chemotaxis to Listeria monocytogenes in neurolisteriosis depends on microglia-released rather than bacterial factors
Source: J Neuroinflammation. 2022 Dec 16;19:304. doi: 10.1186/s12974-022-02653-1 (PMC9758797; doi:10.1186/s12974-022-02653-1)
Supplement: Supplementary file 10 — Additional file 10: Table S3. Conditions tested in the Transwell chemotaxis assay. [file 12974_2022_2653_MOESM10_ESM.pdf]

Table S3 – Conditions tested in the Transwell chemotaxis assay

Condition tested	Concentration/dilution
Bovine IL-8[a]	50 ng/ml
fMLP[b]	0.1 μM & 1 μM
fMIVIL[c]	1 nM & 10 nM
fMIVTLF[c]	1 nM & 10 nM
WT-*Lm*[d]	6x10^5 CFU/well
Under-agarose WT-*Lm*	6x10^5 CFU/well
Heat-killed WT-*Lm*	6x10^5 CFU/well
WT-*Lm* culture supernatant	1:10
Serum-opsonized WT-*Lm*	6x10^5 CFU/well
EGDe-*Lm*[e]	6x10^5 CFU/well
Δ*hly*-*Lm*[f]	6x10^5 CFU/well
Astrocyte-conditioned medium	undiluted
Infected astrocyte-conditioned medium	undiluted
FBBC-1-conditioned medium	undiluted
Infected FBBC-1-conditioned medium	undiluted
Microglia-conditioned medium	undiluted
Infected microglia-conditioned medium	undiluted

[a] Kingfisher Biotech, Inc. St. Paul, MN, USA

[b]N-Formyl-Met-Leu-Phe (Sigma Aldrich Chemie Ltd., St. Louis, USA)

[c]Synthesized at the Institute of Chemistry and Biotechnology, Wädenswil, Switzerland

[d]*Lm* strain JF5203 (lineage I, clonal complex 1, sequence type 1, https://www.ncbi.nlm.nih.gov/nuccore/NZ_LT985474.1)

[e]*Lm* EGDe strain (serotype 1/2A, ATCC BAA-679)[34]

[f]Δ*hly-Lm* was generated in this study (see Supplementary methods 1).
